# The deubiquitinating enzyme UCHL3 promotes bladder cancer progression and glycolysis through enhancing PKM2 protein stability

**DOI:** 10.3389/fonc.2026.1883763

**Published:** 2026-09-18

**Authors:** Gengsheng Shi, Yanyan Wu, Yingying Wang, Suyu Wang, Wan Xiang, Wenqin Yin, Ruirui Li

**Affiliations:** 1 Department of Clinical and Public Health, Jiangsu College of Nursing, Huaian, Jiangsu, China; 2 Department of Endocrinology, Taizhou Second People’s Hospital Affiliated to Yangzhou University, Taizhou, Jiangsu, China; 3 Endoscopy Center, Jiangsu Province (Suqian) Hospital, Suqian, Jiangsu, China; 4 Department of Biological Repositories, Human Genetic Resources Preservation Center of Hubei Province, Hubei Key Laboratory of Urological Diseases, Zhongnan Hospital of Wuhan University, Wuhan, China; 5 Medical Department, Jiangsu Province (Suqian) Hospital, Suqian, Jiangsu, China

**Keywords:** bladder cancer, glycolysis, PKM2, ubiquitination, UCHL3

## Abstract

Pyruvate kinase M2 (PKM2) is a critical rate-limiting enzyme in glycolysis and is frequently upregulated in bladder cancer (BCa), contributing to the Warburg effect and tumor progression. However, the mechanism supporting abnormal PKM2 stabilization via deubiquitination in BCa remains to be characterized. Here, we identified ubiquitin C-terminal hydrolase L3 (UCHL3) as a bona fide deubiquitylase of PKM2 in BCa. UCHL3 stabilized PKM2 in a deubiquitylation activity-dependent manner. UCHL3 depletion significantly decreased PKM2 protein levels, suppressed glycolytic activity, and inhibited BCa cell proliferation, colony formation, migration *in vitro*, as well as xenograft tumor growth *in vivo*. UCHL3 knockdown accelerated PKM2 proteasomal degradation, while overexpression of wild-type UCHL3—but not the catalytically inactive C95A mutant—directly interacted with PKM2, removed K48-linked ubiquitin chains, and prolonged its half-life. Clinically, UCHL3 was markedly upregulated in BCa tissues and cell lines compared with normal urothelium, correlated with higher tumor grade, and was associated with poor patient prognosis; its expression also positively correlated with PKM2 levels. In general, our results demonstrated that UCHL3 plays a pivotal role in stabilizing PKM2, which in turn facilitates glycolysis and tumorigenesis in BCa, suggesting that UCHL3 may prove to be a potential target for the treatment of BCa.

## Introduction

Bladder cancer (BCa) is a frequent and aggressive malignancy and constitutes one of the most common tumors of the genitourinary system. Epidemiological evidence indicates that BCa is the ninth most prevalent cancer worldwide, accounting for approximately 613,000 new diagnoses and 220,000 deaths (1). Clinically, nearly 75% of newly identified cases present as non–muscle-invasive bladder cancer (NMIBC), whereas the remaining patients are diagnosed with muscle-invasive bladder cancer (MIBC). Among NMIBC cases, about 69% are low grade, while muscle-invasive or metastatic disease is predominantly high grade (2). NMIBC is characterized by substantial clinical burden, with a 5-year recurrence rate of 31%–78% and a progression rate ranging from 0.93% in low-risk groups to 40% in very high-risk groups. For treated MIBC, the 5-year overall survival is approximately 48%, but this rate declines to ~5.5% once metastasis occurs (1, 3). Accordingly, delineation of the molecular basis of malignant progression and the development of more effective therapeutic strategies remains urgent.

Metabolic reprogramming represents a defining feature of cancer, enabling tumor cells to maintain rapid growth and persist under adverse microenvironmental conditions. In BCa, glycolysis is frequently sustained despite the presence of oxygen (the Warburg effect), thereby supporting bioenergetic needs, biosynthetic precursor generation, and redox homeostasis (4). Pyruvate kinase M2 (PKM2), a key rate-limiting enzyme in glycolysis, catalyzes the final, rate-limiting step of glycolysis, promoting ATP production while preserving glycolytic flux (5). PKM2 is broadly overexpressed across malignancies, including BCa, and is associated with tumor progression, enhanced aggressiveness, and metabolic adaptation (6). The abundance and function of PKM2 are modulated by post-translational modifications, including phosphorylation and ubiquitination, largely through the ubiquitin–proteasome system, which regulates enzymatic activity and proteolytic turnover (7). Deubiquitinases (DUBs) counteract protein ubiquitination and serve essential roles in stabilizing substrates and coordinating diverse cellular programs (8, 9). Although growing evidence supports the involvement of DUBs in tumor metabolic regulation, the specific DUBs and mechanistic pathways governing PKM2 stability in BCa remain insufficiently characterized. A interrogation of the interplay between metabolic control and proteolytic regulation may therefore provide mechanistic insight relevant to tumor biology, therapeutic discovery, and precision medicine.

While PKM2 has been widely implicated in enhancing glycolytic flux and promoting tumor progression across cancer types (5), the upstream DUB-dependent deubiquitination and stabilization mechanisms—particularly in BCa—are still poorly understood. Existing work has predominantly emphasized PKM2 phosphorylation, whereas the dynamic balance between PKM2 ubiquitination and deubiquitination has received comparatively limited attention, potentially leaving key aspects of metabolic regulation unresolved (10, 11). Moreover, although several glycolytic enzyme inhibitors show antitumor activity in preclinical settings, therapeutic performance is often constrained by pathway compensation and off-target liabilities, reinforcing the need to identify upstream regulators of PKM2 that may be more tractable targets. In BCa, elevated PKM2 expression is linked to unfavorable clinicopathological characteristics (12, 13). Nevertheless, the contribution of DUBs—such as the cysteine protease family member Ubiquitin C-terminal Hydrolase L3 (UCHL3)—to the regulation of glycolytic enzymes and malignant progression in BCa has not been systematically examined (6). UCHL3 is aberrantly expressed in multiple cancer types (5), yet its specific role in BCa glycolysis and malignant phenotypes remains unclear; addressing this gap may clarify how proteolytic stability interfaces with metabolic reprogramming.

In this study, we sought to define the molecular mechanism by which UCHL3 regulates PKM2 stability via deubiquitination and to determine how this regulatory axis influences BCa glycolytic activity and tumor progression. We posited that UCHL3 interacts directly with PKM2, suppresses PKM2 ubiquitination, increases PKM2 protein stability, and activates downstream metabolic pathways, thereby promoting BCa cell proliferation and migration. To evaluate this model, BCa cell models and clinical specimens were analyzed using integrated molecular, biochemical, and cellular approaches to characterize the structural basis, enzyme dependence, and phenotypic consequences of the UCHL3–PKM2 axis. Glycolytic outputs, including extracellular acidification rate and lactate production, were quantified, and public databases together with tissue-based analyses were used to examine associations between UCHL3 expression, clinicopathological parameters, and patient prognosis. Collectively, these findings support UCHL3 as a potential metabolic therapeutic target in BCa and provide a mechanistic rationale for strategies aimed at disrupting metabolic reprogramming in this disease.

## Materials and methods

### Cell culture and transfection

SV-HUC-1, T24, 5637, and RT-4 cells were cultured in RPMI-1640 medium, whereas J82 cells were grown in minimum essential medium (MEM). HEK293T cells were cultured in Dulbecco’s modified Eagle medium (DMEM). All culture media contained 10% fetal bovine serum (FBS). The cell lines were sourced from the Cell Bank of the Chinese Academy of Sciences (Shanghai, China) and incubated at 37 °C in a humidified atmosphere with 5% CO_2_. All cell lines used in this study were authenticated by short tandem repeat profiling and tested negative for mycoplasma contamination prior to use in experiments.

### Deubiquitinase siRNA library screening

An ON-TARGETplus siRNA library targeting deubiquitinases was acquired from Dharmacon (Catalog No. G-104705-025, Dharmacon, Lafayette, CO, USA). An exploratory pilot screen comprising 24 DUB-related targets was performed in T24 cells using pooled ON-TARGETplus siRNAs. The screen was conducted in three replicate experiments. Cells were harvested 48 h after transfection, and PKM2 protein abundance was assessed by Western blotting. PKM2 densitometric signals were normalized to β-actin and subsequently to the matched siControl within each experiment. Because the screen was hypothesis-generating, no formal numerical cutoff was prespecified. UCHL3 was prioritized because its depletion produced the strongest and most reproducible reduction in PKM2 abundance. On-TARGETplus siRNAs are designed and chemically modified to reduce off-target effects while maintaining strong silencing efficacy for high-confidence screening (9, 14). The complete screening dataset is provided in **Supplementary Table 4**.

### siRNAs and plasmids

Plasmids encoding PKM2, wild-type (WT) UCHL3, and the catalytically inactive UCHL3 mutant were purchased from Hanbio (Shanghai, China). The Myc-K6, -K11, -K27, -K29, -K33, -K48, -K63, and -Ub plasmids were acquired from Addgene. siRNAs targeting UCHL3 were synthesized by GenePharma (Shanghai, China). Plasmids and siRNAs were transfected using Lipofectamine 3000 (L3000015, Invitrogen). When cells reached 70%–90% confluence in 6-well plates, plasmid transfection was performed as follows: plasmids were diluted in 100 μL Opti-MEM (Tube 1), P3000 reagent was added (Tube 2), and the mixtures were combined and incubated at room temperature for 15 min. DNA–lipid complexes were then added to cells and incubated at 37 °C for 2–3 days. For siRNA transfection, the same procedure was used except that P3000 reagent was not added during siRNA dilution. siRNA sequences are provided in **Supplementary Table 1**.

### Antibodies

Sources, catalog numbers, and working dilutions for all primary and secondary antibodies used in this study are summarized in **Supplementary Table 2**.

### MTT assay

Treated cells were seeded into 96-well plates at a density of 2 × 10³ cells per well and cultured at 37 °C for 24 h. MTT solution (20 μL; HY-15924, MCE) was added to each well and incubated for 4 h, after which the supernatant was removed. DMSO (160 μL) was added and plates were shaken for 10 min to fully dissolve formazan crystals. Absorbance was measured using a microplate reader. Cell proliferation was assessed daily for 5 consecutive days under identical conditions.

### Colony formation assay

Treated cells were seeded into 6-well plates at 1 × 10³ cells per well and incubated at 37 °C for 1–2 weeks until visible colonies formed. Medium was removed and cells were washed with PBS, fixed with 4% paraformaldehyde for 4 h, and stained with 0.1% crystal violet for 4 h. Plates were gently rinsed with distilled water, air-dried, photographed, and colonies were counted.

### Transwell migration assay

The lower chamber of Transwell inserts was filled with complete medium containing 10% FBS as a chemoattractant. Transfected cells (3 × 10^4^ per well) were suspended in serum-free medium and seeded into the upper chamber. After 24 h, medium was removed and non-migrated cells on the upper surface of the membrane were gently wiped off. Migrated cells on the lower surface were fixed with 4% paraformaldehyde for 4 h, stained with 0.1% crystal violet for 4 h, rinsed with distilled water, air-dried, and counted in randomly selected microscopic fields.

### RNA extraction and qRT-PCR

RNA extraction and quantitative reverse transcription polymerase chain reaction (RT-qPCR).

Total RNA was isolated from cells with the Hipure Total RNA Mini Kit (Magen, Guangzhou, China). Reverse transcription to cDNA used the RevertAid First Strand cDNA Synthesis Kit (Thermo Fisher Scientific, Vilnius, Lithuania) per the manufacturer’s guidelines. RT-qPCR reactions (20 μL) employed SYBR Green reagents (Toyobo, Japan) on a 7500 Fast Real-Time PCR System (Applied Biosystems, Foster City, CA, USA), with 0.2 μM of each primer. Thermal cycling consisted of 95 °C for 3 min, followed by 40 cycles of 95 °C for 10 s and 60 °C for 30 s, then a melting curve (65–95 °C) to verify specificity. Primers were designed to produce amplicons of approximately 80–200 bp, with Tm values around 58–62 °C and minimal self-complementarity; exon–exon junctions were preferred when feasible. Specificity was confirmed by a single melting peak. All primers were synthesized by Tsingke (Wuhan, China). GAPDH served as the internal control, and relative expression levels were calculated via the 2^^-^ΔΔCt^ method. Primer sequences and annealing temperatures are provided in **Supplementary Table 3**.

### Extracellular acidification rate measurement

Glycolytic activity was evaluated by measuring extracellular acidification rate (ECAR) using the Seahorse XF Glycolysis Stress Test Kit (103020-100, Seahorse Bioscience) on XFe96 cell culture microplates (101085-004, Seahorse Bioscience). Briefly, 2,000 cells were seeded and incubated at 37 °C with 5% CO_2_ for 12 h. Medium was then replaced with XF base medium, and cells were incubated for 1 h in a non-CO_2_ incubator. Glucose (10 mM), oligomycin (1 μM), and 2-deoxy-D-glucose (2-DG, 50 mM) were injected sequentially. Parameters including basal glycolysis, glycolytic capacity, and glycolytic reserve were recorded. ECAR values were normalized to protein content per well.

### Immunohistochemistry

The human bladder cancer tissue microarray HBlaU079Su01 was obtained from Shanghai Outdo Biotech Co., Ltd. Animal tumor tissues were fixed in 10% formalin, dehydrated through graded ethanol, embedded in paraffin, and sectioned. After deparaffinization and rehydration, sections underwent antigen retrieval and were treated with a blocking reagent to suppress endogenous peroxidase activity. Primary antibodies (**Supplementary Table 2**) were applied overnight at 4 °C, followed by incubation with HRP-conjugated secondary antibodies (1:500) for 1 h at room temperature. Color was developed with 3,3′-Diaminobenzidine (DAB) substrate, and sections were counterstained with hematoxylin for 2 min at room temperature. Imaging was performed on a multifunctional scanning microscope (Aperio VERSA8, Leica, Wetzlar, Germany). UCHL3 staining scores were computed by multiplying intensity (0 = negative, 1 = weak, 2 = moderate, 3 = strong) by the proportion of positive cells (0 = negative, 1 = 1%–25%, 2 = 26%–50%, 3 = 51%–75%, 4 = 76%–100%).

### Immunofluorescence

Sterile 22 × 22 mm coverslips were placed in 6-well plates, and cells were seeded onto coverslips and cultured for 24 h before plasmid transfection. After an additional 24 h, cells were fixed with 4% paraformaldehyde, permeabilized with 0.4% Triton X-100, and blocked with 2% bovine serum albumin. Cells were incubated with primary antibodies at 4 °C overnight, followed by fluorophore-conjugated secondary antibodies for 1 h at room temperature in the dark. Nuclei were stained with DAPI (0.5 μg/mL) for 8 min, washed three times with distilled water, and images were captured using a laser confocal microscope (Nikon, Japan).

### Protein stability assay

For proteasome inhibition experiments, cells were treated with MG132 (10 μM) for 4 hours prior to lysis. For CHX chase experiments, cells were treated with cycloheximide (50 µg/mL; HY-12320, MedChemExpress, Monmouth Junction, NJ, USA) and collected at designated time points. PKM2 protein levels were quantified using ImageJ software via grayscale scanning and normalized against a loading control to estimate the relative half-life.

### Animal experiments

PKM2 shRNA is generated by inserting 5′-TTATTTGAGGAACTCCGCCGC-3′ into pLKO.1-puro targeting exon 10 of the PKM2 transcript, as previously described (15), while its control shRNA is generated by inserting 5′-TTCTCCGAACGTGTCACGT-3′ into pLKO.1-puro. T24 cells were transduced with lentiviral vectors (GenePharma, Shanghai, China) to generate stable UCHL3 knockdown (shUCHL3) and control (shNC) cell lines, followed by puromycin selection (1 μg/mL, Sigma). Four-week-old male BALB/c nude mice were purchased from Beijing Shenglige Experimental Animal Technology Co., Ltd. (Beijing, China). All mice were housed under specific pathogen-free (SPF) conditions in a controlled environment (temperature: 20–24 °C; relative humidity: 30–70%; 12-h light/dark cycle) and provided with unrestricted access to food and water at the Animal Experiment Center of Zhongnan Hospital of Wuhan University. The mice were acclimated for one week, and randomly assigned to shNC (n =4) and shUCHL3 (n =4) groups. A xenograft model was established by subcutaneously injecting 150 μL of a PBS solution containing 2 × 10^7^ cells into the dorsal aspect of the mice’s forelimbs. Tumor size was measured at regular intervals using a caliper and calculated according to the formula: Tumor Size = Length × Width² × 0.5 (mm³). In accordance with the regulations of the Institutional Animal Care and Use Committee at Zhongnan Hospital of Wuhan University, the maximum tumor length was not permitted to exceed 20 mm, and the tumor volume was not permitted to exceed 2000 mm³; consequently, none of the experiments conducted in this study exceeded these limits. Mice were euthanized via cervical dislocation, and the tumors were harvested. After weighing, the tumor tissues were fixed in 4% PFA in preparation for subsequent staining.

### Immunoblotting

Cells were lysed in RIPA buffer supplemented with PMSF and phosphatase inhibitors and incubated on ice for 30 min. After adding 5× loading buffer, lysates were denatured in boiling water at 100 °C. Proteins were separated by SDS-PAGE and transferred to PVDF membranes, which were blocked with 5% nonfat milk, incubated with primary antibodies followed by HRP-conjugated secondary antibodies, and visualized by chemiluminescence. Bands were recorded using the BioSpectrum 515 imaging system (UVP).

### Co-immunoprecipitation

Cells were lysed in NP-40 lysis buffer containing protease inhibitors. Total cell lysates were pre-cleared with rabbit IgG for 2 h, then incubated with specific antibodies overnight at 4 °C with rotation. Protein A/G agarose beads (Santa Cruz) were added and incubated for 2 h at 4 °C. Immunocomplexes were washed three times and analyzed by SDS-PAGE and Western blotting.

### *In vivo* deubiquitination assay

HEK293T cells were co-transfected with Myc-Ub, FLAG-PKM2, HA-UCHL3, and HA-UCHL3^C95A^ for 24 h, followed by incubation with 10 μM MG132 for 6 h. Cell lysates were subjected to immunoprecipitation with anti-FLAG antibody and analyzed by immunoblotting.

### Statistical analysis

Experiments were conducted with a minimum of three independent biological replicates unless noted otherwise. Animal group sizes are specified in figure legends. ImageJ software (NIH, v1.53) quantified Western blot bands and cell counts (26). IHC optical density was measured with Image-Pro Plus (v6.0). Survival analyses used R (v4.1.3), while other statistics were performed in GraphPad Prism (v9). Given modest sample sizes, parametric tests were applied when suitable and non-parametric Mann-Whitney U tests for ordinal data such as IHC scores. Log-rank tests compared survival curves. Two-group differences were evaluated by two-tailed unpaired Student’s t-tests; multi-group comparisons employed one-way ANOVA with Dunnett’s post-test.

## Results

### Silencing UCHL3 reduces PKM2 protein expression

Analysis of The Cancer Genome Atlas (TCGA) data revealed that PKM2 is significantly overexpressed in bladder cancer (BCa) tissues compared with adjacent normal bladder tissues (**Supplementary Figure 2A**). This overexpression is further associated with advanced tumor stage (**Supplementary Figure 2B**), non-papillary histological subtype (**Supplementary Figure 2C**), and lymph node metastasis (**Supplementary Figure 2D**). Kaplan–Meier survival analysis indicated that high PKM2 expression predicts unfavorable prognosis in BCa patients (**Supplementary Figure 2E**). To identify DUBs that regulate PKM2 deubiquitination and stabilization in BCa, we constructed an siRNA library targeting multiple DUBs and performed systematic screening in T24 cells. We identified that UCHL3 knockdown markedly reduced PKM2 protein levels (**Figures 1A, B**). To further validate this observation, two independent siRNAs targeting UCHL3 were transfected into T24 and 5637 cells. In both cell lines, UCHL3 depletion consistently decreased PKM2 protein expression (**Figure 1C**). In contrast, RT–PCR analysis showed that UCHL3 knockdown had no effect on PKM2 mRNA abundance in T24 or 5637 cells (**Figures 1D, E**). Conversely, ectopic overexpression of UCHL3 increased PKM2 protein levels in a dose-dependent manner (**Figure 1F**). These results indicate that UCHL3 promotes PKM2 expression at the post-transcriptional level, likely by regulating protein stability rather than transcription.

**Figure 1:**
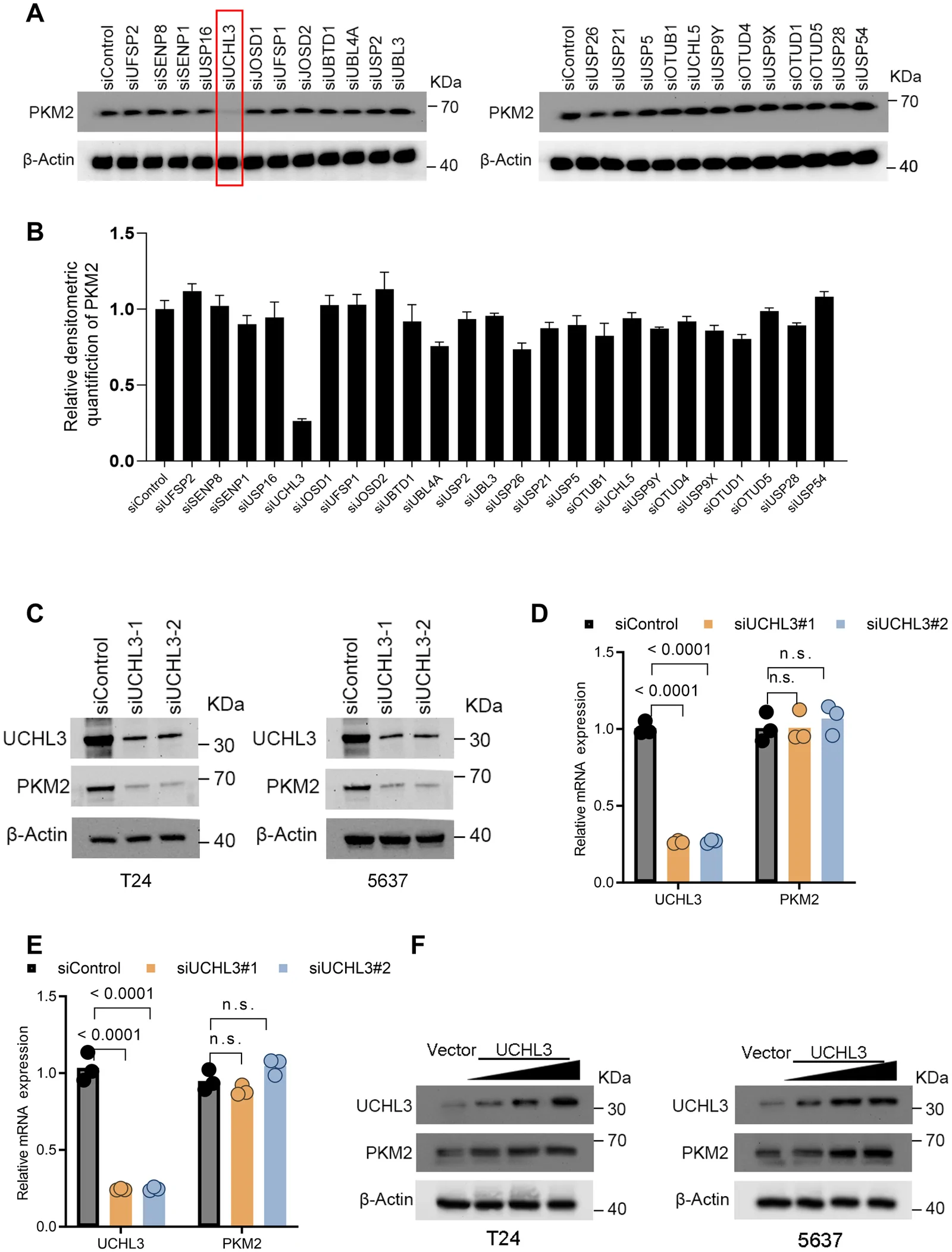
UCHL3 regulates PKM2 protein levels in bladder cancer cells. **(A, B)** siRNAs targeting individual deubiquitinases were introduced into T24 cells. After 48 h, cells were lysed and PKM2 protein levels were analyzed by Western blotting. **(C)** T24 and 5637 cells were transfected with two independent siRNAs targeting UCHL3 (siUCHL3#1/#2) or siControl. UCHL3 and PKM2 protein levels were assessed by Western blotting. Compared with siControl, PKM2 protein levels were markedly decreased in UCHL3-depleted cells. **(D, E)** qRT-PCR analysis of PKM2 mRNA levels in T24 and 5637 cells after UCHL3 knockdown; GAPDH served as an internal control. No significant difference in PKM2 mRNA abundance was observed between siControl and siUCHL3 groups (n = 3 per group). **(F)** T24 and 5637 cells were transfected for 36 h with increasing doses (0.5 μg, 1 μg, 2 μg) of UCHL3 overexpression plasmid. Endogenous PKM2 levels were determined by immunoblotting using anti-PKM2 antibody. PKM2 protein increased in a dose-dependent manner upon UCHL3 overexpression, with no obvious change in vector controls. Data are presented as mean ± SD.

### UCHL3 stabilizes PKM2 through its deubiquitinase activity

We next investigated whether UCHL3 regulates PKM2 stability through deubiquitination. UCHL3 knockdown in T24 and 5637 cells significantly reduced PKM2 protein levels, and the proteasome inhibitor MG132 partially reversed this effect (**Figures 2A, B**), whereas the autophagy inhibitor chloroquine (CQ) did not. These results suggest proteasome-dependent PKM2 degradation. Overexpression of wild-type UCHL3 (UCHL3-WT) markedly increased PKM2 protein levels, whereas the catalytically inactive mutant (UCHL3-C95A) had little effect (**Figures 2C, D**), indicating that UCHL3 deubiquitinase activity is essential for PKM2 stabilization.

**Figure 2:**
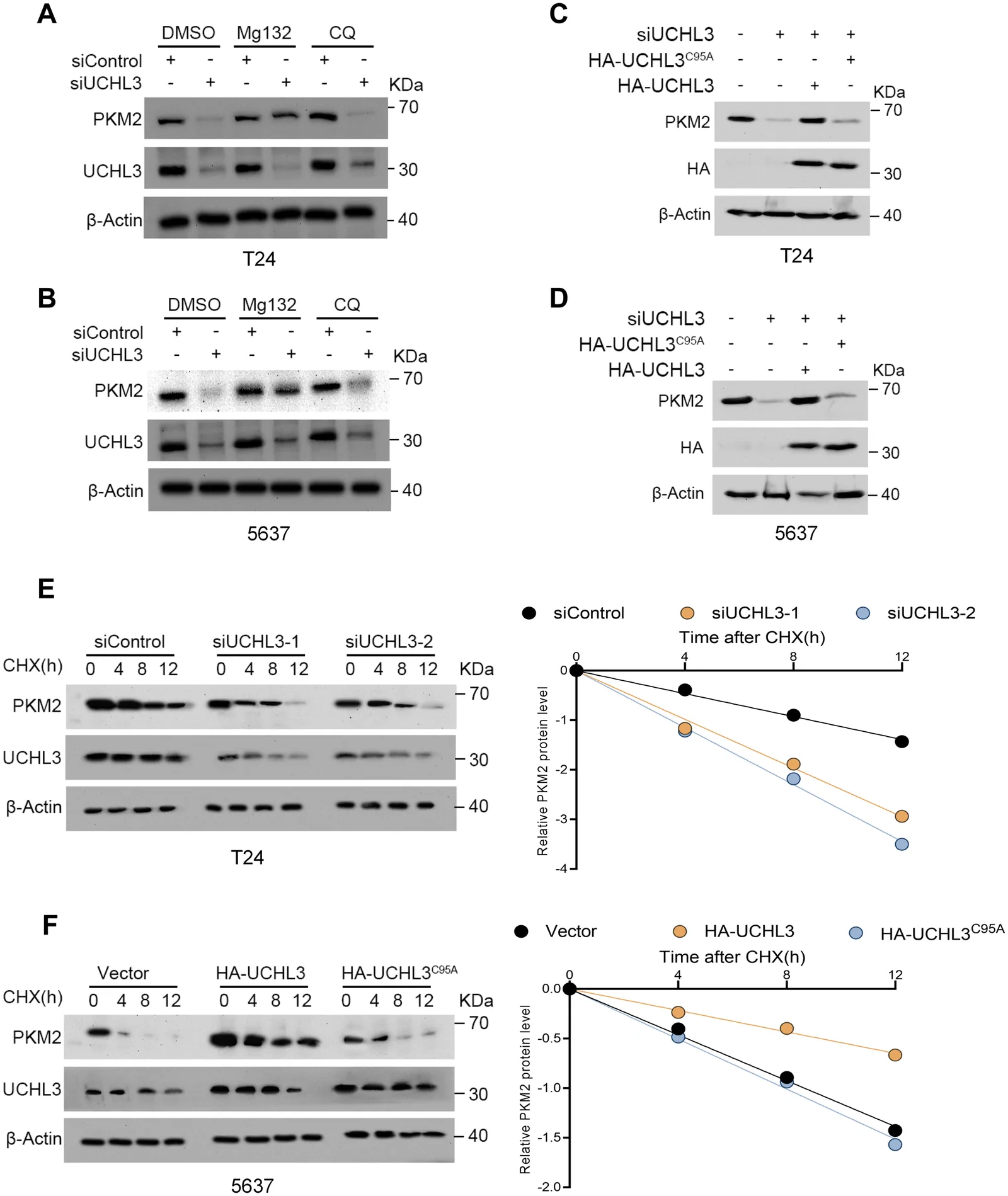
UCHL3 stabilizes PKM2 via its deubiquitinase activity. **(A, B)** T24 and 5637 cells were transfected with siControl or siUCHL3, and after 24 h were treated with DMSO, 10 μM MG132 (#S2619, Selleck), or 20 μM chloroquine (CQ; #S8808, Selleck) for 8 h. PKM2 protein levels were examined by immunoblotting. Western blotting showed that UCHL3 depletion reduced PKM2 protein, and MG132 partially restored PKM2 expression. **(C, D)** T24 and 5637 cells were transfected with wild-type UCHL3 (UCHL3-WT), catalytically inactive UCHL3-C95A, or empty vector. PKM2 levels were analyzed by Western blotting. UCHL3-WT markedly increased PKM2 protein, whereas UCHL3-C95A had minimal effect, indicating that UCHL3 deubiquitinase activity is essential for PKM2 stabilization. **(E)** CHX chase assays were performed in T24 and 5637 cells transfected with siControl or siUCHL3, and PKM2 levels were monitored at the indicated time points. PKM2 degradation was accelerated and its half-life shortened in UCHL3-deficient cells. **(F)** CHX chase assays were conducted in cells overexpressing UCHL3-WT, UCHL3-C95A, or empty vector. Compared with vector and UCHL3-C95A, UCHL3-WT significantly prolonged the half-life of PKM2. Data are presented as mean ± SD; n = 3 independent replicates for **(E, F)**. Because endogenous UCHL3 and exogenous UCHL3-WT/C95A have similar molecular weights, the anti-UCHL3 band represents the total UCHL3 signal and was not used to distinguish the individual UCHL3 forms.

To directly assess the impact of UCHL3 on PKM2 stability, we performed cycloheximide (CHX) chase assays. In UCHL3-deficient T24 cells, PKM2 degradation was accelerated and its half-life was shortened (**Figure 2E**). UCHL3-WT overexpression significantly prolonged PKM2 half-life in 5637 cells, whereas UCHL3-C95A had no obvious effect (**Figure 2F**). Together, these data demonstrate that UCHL3 stabilizes PKM2 by inhibiting proteasome-dependent degradation via its deubiquitinase activity, thereby acting as a key regulator of glycolysis.

### UCHL3 directly binds PKM2 to mediate deubiquitination

To define subcellular localization and interaction between UCHL3 and PKM2, we first performed immunofluorescence staining in T24 and 5637 cells. Confocal microscopy revealed that endogenous UCHL3 and PKM2 predominantly co-localize in the cytoplasm (**Figure 3A**). We then co-transfected exogenous UCHL3 and PKM2 into 293T cells and performed co-immunoprecipitation (Co-IP), detecting both proteins in the same complex (**Figure 3B**). Moreover, endogenous PKM2 was detectable in T24 and 5637 cells upon immunoprecipitation of endogenous UCHL3 (**Figure 3C**). Given that UCHL3 is a canonical DUB, we next assessed its effect on PKM2 ubiquitination. In the presence of MG132, UCHL3 knockdown significantly increased PKM2 ubiquitination (**Figure 3D**). Conversely, overexpression of UCHL3-WT but not UCHL3-C95A significantly reduced PKM2 ubiquitination (**Figure 3E**). To investigate the specific type of ubiquitin chain targeted by UCHL3 during the deubiquitination of PKM2, we further conducted ubiquitination assays utilizing a series of ubiquitin mutants. The results revealed that UCHL3 effectively removes K48-linked ubiquitin chains from PKM2 (**Figure 3F**). Collectively, these results indicate that UCHL3 directly removes K48 ubiquitin chains from PKM2 through its deubiquitinase activity, thereby suppressing proteasome-mediated PKM2 degradation.

**Figure 3:**
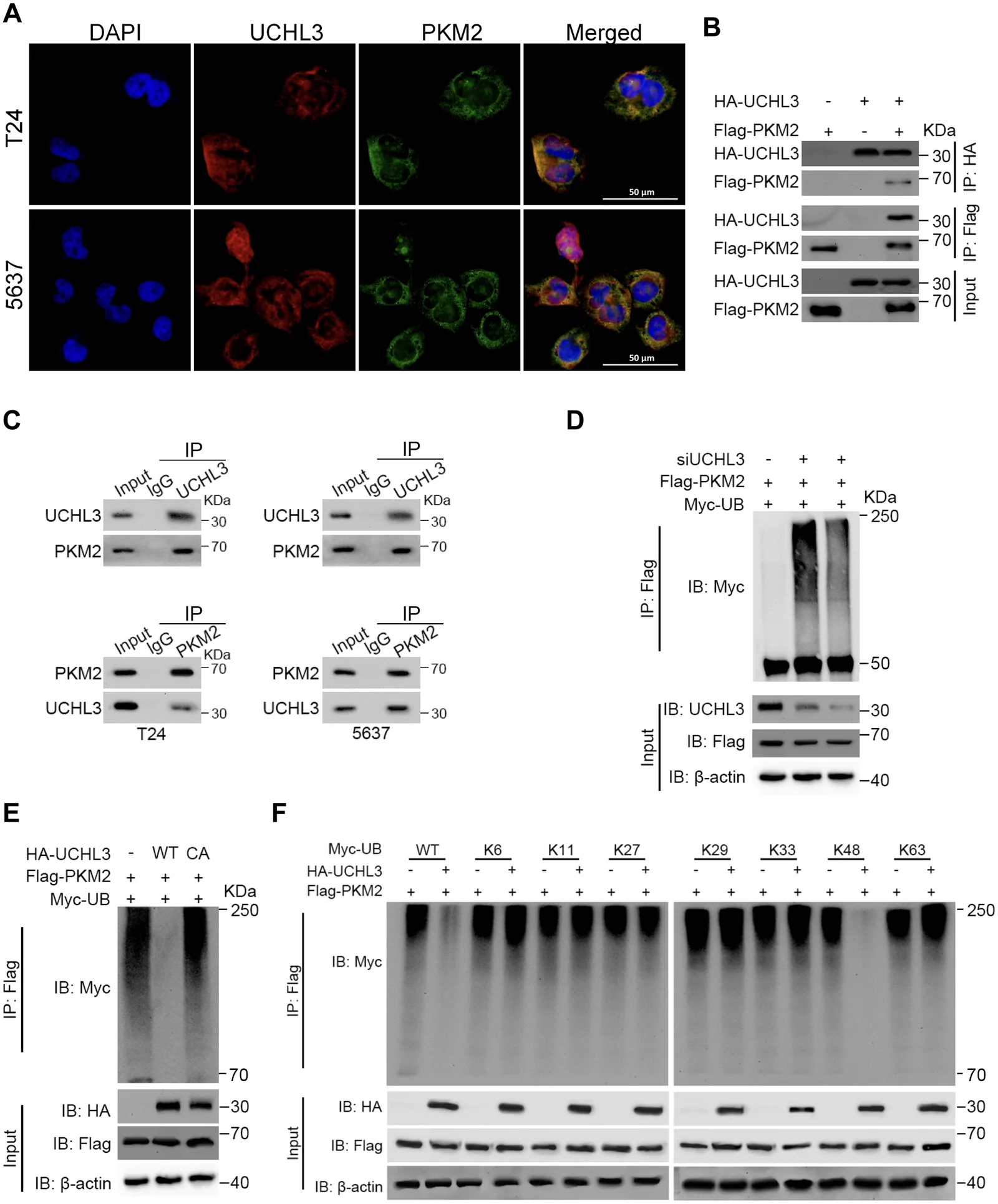
UCHL3 directly interacts with PKM2 and mediates its deubiquitination. **(A)** Immunofluorescence staining in BCa cells was used to assess subcellular localization of endogenous UCHL3 and PKM2; nuclei were stained with DAPI. Confocal imaging showed predominant cytoplasmic co-localization of UCHL3 and PKM2. **(B)** HEK293T cells were co-transfected with HA-UCHL3 and Flag-PKM2 constructs and subjected to Co-IP. Immunoprecipitation with anti-HA or anti-Flag followed by Western blotting confirmed complex formation between exogenous UCHL3 and PKM2. **(C)** Immunoblotting of UCHL3-IP or IgG-IP in T24 and 5637 cells; 10% of the input lysate used for IP is shown. **(D)** Flag-PKM2, Myc-Ub, and siUCHL3 were introduced into T24 cells for 48 h, followed by MG132 treatment for 6 h. Lysates were subjected to anti-Flag Co-IP and analyzed by Western blotting with anti-Myc antibody. **(E)** HA-UCHL3-WT or HA-UCHL3-C95A, Flag-PKM2, and Myc-Ub were introduced into HEK293T cells, followed by incubation with 10 μM MG132 for 6 h. Lysates were subjected to anti-Flag Co-IP and analyzed by Western blotting with anti-Myc antibody. **(F)** Myc-WT, K6, K11, K27, K29, K33, K48 or K63 Ub were co-transfected with Flag-PKM2 and Myc-UCHL3 into HEK293T cells. After treatment with 10 μM MG132 for 6 h, cell lysates were subjected to ubiquitination assay and the ubiquitination level of PKM2 detected by Flag antibody.

### UCHL3 expression correlates with bladder cancer progression and prognosis

Given the critical role of UCHL3 in regulating PKM2 stability, we further examined its expression pattern and clinical relevance in BCa. TIMER2 database analysis showed that UCHL3 expression exhibits tissue-specific differences across urinary system cancers, including BCa, KICH, KIRC, and PRAD; notably, UCHL3 was significantly upregulated only in BCa, suggesting potential BCa-specific functions (**Supplementary Figure 1A**). Besides, UCHL3 expression correlates with advanced tumor stage (**Supplementary Figure 1C**), non-papillary histology (**Supplementary Figure 1D**), and lymph node metastasis (**Supplementary Figure 1E**). In the TCGA–GTEx dataset (normal, n = 28; tumor, n = 407), UCHL3 mRNA levels were broadly elevated in BCa tissues (**Figure 4A**). Compared with normal urothelial cells, BCa cell lines displayed higher UCHL3 mRNA expression (**Figure 4B**). We next evaluated UCHL3 protein expression by immunohistochemistry (IHC) in the HBlaU079Su01 tissue microarray containing paired adjacent non-tumor tissues (n = 15) and BCa tissues (n = 57). UCHL3 protein levels were markedly higher in tumor tissues than in adjacent tissues (**Figures 4C–F**), and were further increased in higher-grade tumors. Prognostically, patients in the GSE13507 cohort were stratified into high- and low-UCHL3 expression groups; Kaplan–Meier analysis showed that high UCHL3 expression was associated with significantly reduced overall survival (P = 0.018; **Figure 4G**). Additionally, UCHL3 and PKM2 mRNA expression levels were positively correlated in the TCGA BCa cohort. Together, these findings suggest that UCHL3 is highly expressed in BCa and is closely linked to disease progression and poor prognosis.

**Figure 4:**
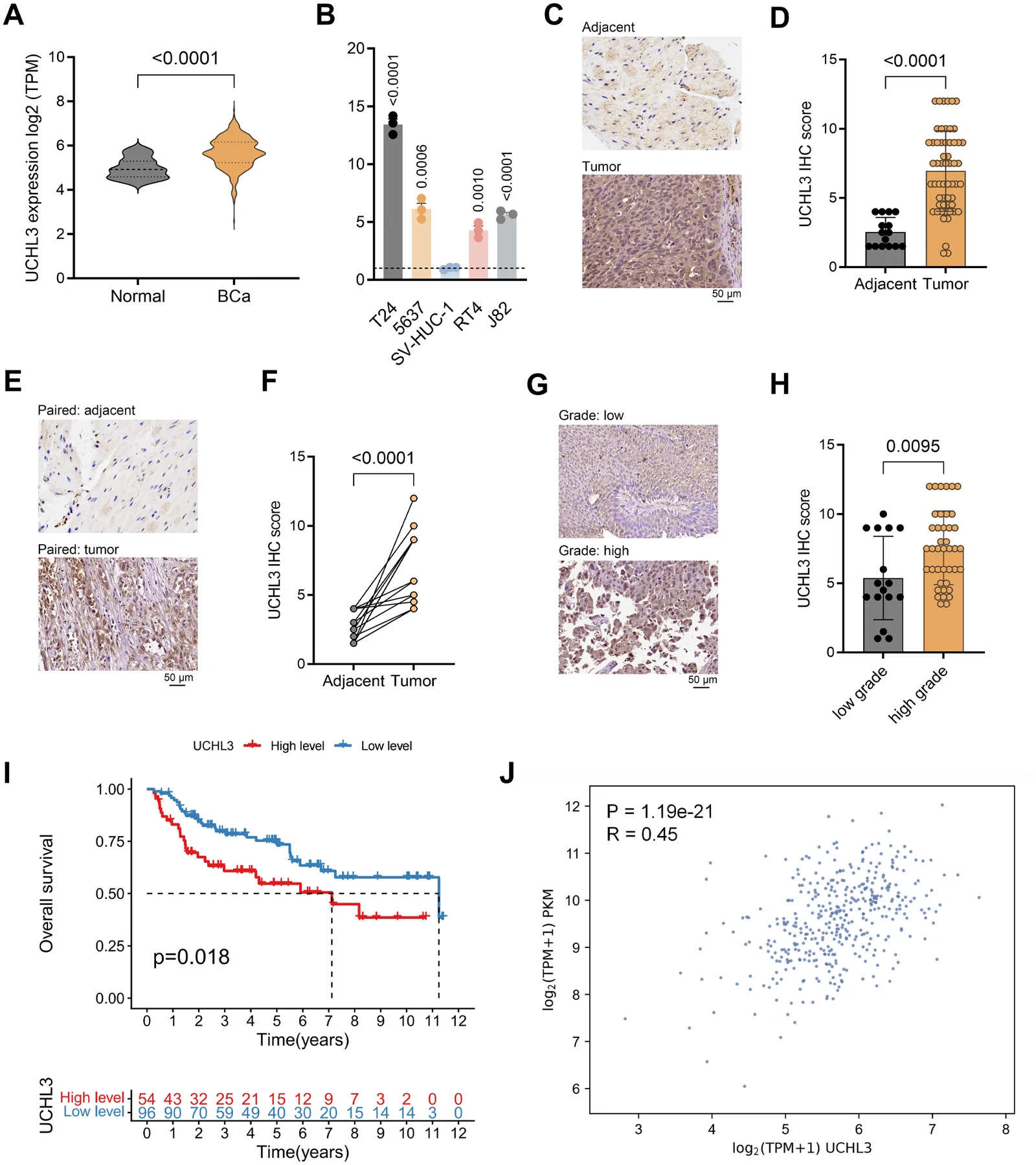
UCHL3 is upregulated in bladder cancer and is associated with high tumor grade and poor prognosis. **(A)** UCHL3 mRNA levels were compared between BCa tissues (tumor, n = 407) and normal bladder tissues (normal, n = 28) using TCGA-GTEx data; UCHL3 was significantly upregulated in BCa. **(B)** qRT-PCR analysis of UCHL3 expression in BCa cell lines (T24, 5637, J82, and RT-4) and immortalized normal urothelial cells (SV-HUC-1) (n = 3 per group). Relative to SV-HUC-1, UCHL3 was elevated in most BCa cell lines, particularly in T24 and 5637. **(C, D)** Representative IHC images **(C)** and quantification **(D)** of UCHL3 protein in adjacent non-tumor tissues (n = 15) and tumor tissues (n = 57) from the HBlaU079Su01 cohort. **(E, F)** Representative paired IHC images **(E)** and quantification **(F)** of UCHL3 in adjacent tissues (n = 15) and matched tumors (n = 15) from the same cohort. **(G, H)** Representative IHC images **(G)** and quantification **(H)** of UCHL3 in low-grade (n = 16) versus high-grade (n = 41) BCa. **(I)** Kaplan–Meier overall survival analysis of the GEO GSE13507 BCa cohort stratified by UCHL3 expression (high vs low). High UCHL3 expression was associated with significantly worse overall survival (P = 0.018, log-rank test). **(J)** Correlation between UCHL3 and PKM2 mRNA expression in the TCGA BCa cohort; scatter plot with regression line indicates a significant positive correlation. Data are presented as mean ± SD.

### UCHL3 knockdown suppresses bladder cancer growth and metastatic potential

We next assessed the impact of UCHL3 on malignant phenotypes in BCa cells. In T24 and 5637 cells, MTT assays showed that UCHL3 knockdown significantly inhibited cell proliferation (**Figures 5A, B**). Colony formation assays consistently revealed fewer and smaller colonies in UCHL3-deficient cells (**Figure 5C**). Transwell migration assays demonstrated that UCHL3 depletion significantly reduced migration of T24 and 5637 cells (**Figure 5D**), indicating that UCHL3 promotes migratory capacity. *In vivo*, we established subcutaneous xenograft models using T24 cells stably expressing shUCHL3 or control shRNA (shNC). Knockdown efficiency was confirmed prior to implantation by qPCR (**Figure 5E**). Compared with the shNC group, tumors derived from shUCHL3 cells exhibited markedly reduced volume and weight and grew more slowly over time (**Figures 5F–H**). Collectively, these *in vitro* and *in vivo* results demonstrate that UCHL3 promotes BCa cell proliferation and metastasis, and that UCHL3 loss robustly suppresses malignant behavior.

**Figure 5:**
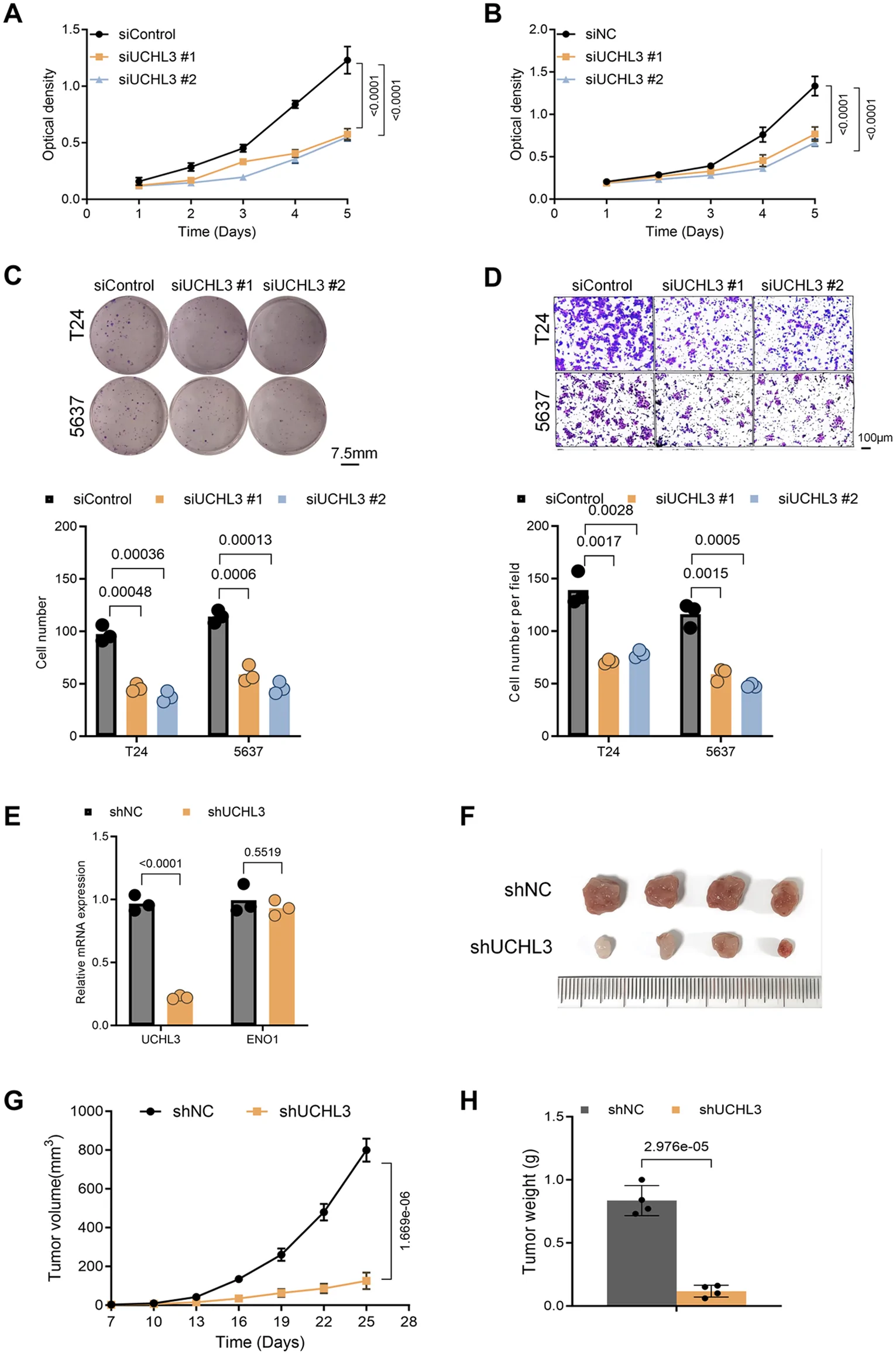
UCHL3 knockdown suppresses malignant phenotypes of bladder cancer cells. **(A, B)** MTT assays were performed for 5 consecutive days in T24 and 5637 cells transfected with siControl or siUCHL3 to evaluate proliferation (n = 5 per group). **(C)** Colony formation assays were used to assess clonogenic capacity. Cells transfected with siControl or siUCHL3 were seeded at low density, cultured for 1–2 weeks, stained with crystal violet, and colonies were counted. Quantification shows that UCHL3 depletion markedly reduced colony numbers (n = 3 per group). **(D)** Transwell migration assays were used to evaluate cell migration (n = 3 per group). Transfected T24 and 5637 cells were seeded in serum-free medium in the upper chamber with serum-containing medium in the lower chamber. After 24 h, migrated cells on the underside of the membrane were fixed, stained, and counted; quantification shows significantly fewer migrated cells upon UCHL3 depletion. **(E)** T24 cells were transduced with lentiviral vectors to generate stable UCHL3 knockdown (shUCHL3) and control (shNC) lines. Knockdown efficiency was validated by qRT-PCR (n = 3 per group). **(F)** Representative images of excised xenograft tumors from nude mice implanted subcutaneously with shNC or shUCHL3 T24 cells (n = 4 per group); tumors were smaller in the shUCHL3 group. **(G)** Tumor growth curves over time; tumors grew significantly more slowly in the shUCHL3 group (n = 4 per group). **(H)** Final tumor weights at endpoint (n = 4 per group), showing reduced tumor mass in the shUCHL3 group. Data are presented as mean ± SD.

### UCHL3 knockdown impairs glycolytic capacity in bladder cancer cells

To investigate the metabolic consequences of UCHL3 dysregulation in bladder cancer (BCa) cells, we first assessed glycolytic function by measuring the extracellular acidification rate (ECAR) using Seahorse XF glycolysis stress tests. The results showed that ectopic overexpression of UCHL3 markedly increased glycolytic flux in BCa cells (**Figure 6A**), suggesting that UCHL3 promotes glucose metabolism toward a more glycolytic phenotype. Consistent with this observation, UCHL3 overexpression also led to a significant elevation in lactate production (**Figure 6B**), further indicating enhanced glycolytic activity and increased conversion of pyruvate into lactate. In contrast, silencing of UCHL3 in T24 and 5637 cells significantly impaired glycolytic performance. Specifically, UCHL3 knockdown reduced basal glycolysis as well as glycolytic capacity, as evidenced by decreased ECAR values in Seahorse assays (**Figures 6C, D**). These findings indicate that endogenous UCHL3 is required to sustain the high glycolytic state of BCa cells. Given that aerobic glycolysis serves as an important source of energy and biosynthetic intermediates in rapidly proliferating tumor cells, the reduction in glycolytic activity caused by UCHL3 depletion may have profound effects on tumor cell viability and growth. In line with the ECAR results, biochemical analyses further demonstrated that UCHL3 depletion significantly reduced intracellular ATP levels (**Figure 6E**), indicating compromised energy production. Moreover, lactate generation was markedly decreased following UCHL3 knockdown (**Figure 6F**), supporting the notion that UCHL3 is essential for maintaining glycolytic output in BCa cells. Conversely, restoration or overexpression of UCHL3 enhanced glycolysis-associated metabolic parameters, reinforcing its role as a positive regulator of tumor metabolic reprogramming.

**Figure 6:**
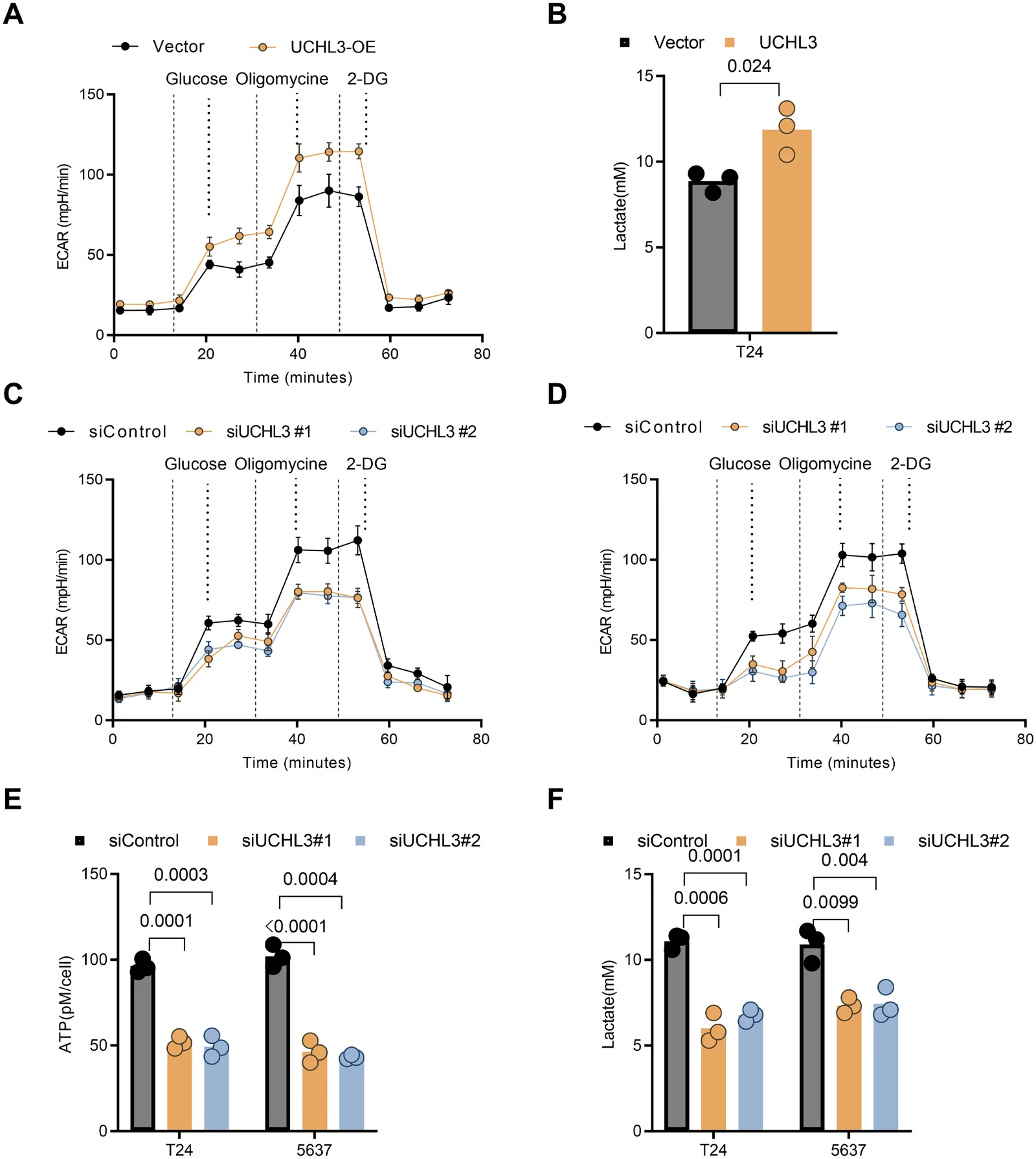
UCHL3 promotes glycolytic metabolism in bladder cancer cells via PKM2. **(A, B)** Seahorse XF glycolysis stress tests in UCHL3-overexpressing T24 cells showed increased ECAR **(A)** and elevated lactate levels **(B)** (n = 5 per group). **(C, D)** Seahorse XF glycolysis stress tests were performed in UCHL3-depleted T24 and 5637 cells to measure ECAR (n = 5 per group). Basal glycolysis and glycolytic capacity were significantly reduced upon UCHL3 knockdown compared with siNC or vector controls. **(E)** Intracellular ATP levels were measured after UCHL3 knockdown (n = 3 per group), showing decreased ATP content. **(F)** Lactate production in culture supernatants was quantified as an indicator of glycolytic output (n = 3 per group); UCHL3 knockdown significantly reduced lactate generation. **(G)** Schematic model of the proposed mechanism. UCHL3 acts as a PKM2 deubiquitinase that stabilizes PKM2 via its deubiquitinating activity, thereby facilitating tumor growth, metastasis, and glycolysis. Data are presented as mean ± SD.

### PKM2 overexpression rescues the inhibitory effects of UCHL3 knockdown on BCa cells

To determine whether PKM2 mediates the oncogenic effects of UCHL3 in BCa cells, rescue experiments were performed by overexpressing PKM2 in UCHL3-knockdown T24 and 5637 cells. MTT assays showed that UCHL3 knockdown suppressed the proliferation of both T24 and 5637 cells, whereas PKM2 overexpression markedly restored cell proliferative capacity (**Figures 7A,B**). Consistently, colony formation assays demonstrated that the reduced clonogenic ability caused by UCHL3 depletion was rescued by PKM2 overexpression (**Figures 7C, D**). Transwell assays further revealed that PKM2 overexpression reversed the inhibitory effect of UCHL3 knockdown on the migratory ability of T24 and 5637 cells (**Figures 7E, F**). Moreover, Seahorse XF Glycolysis Stress Test analysis showed that the decrease in extracellular acidification rate (ECAR) induced by UCHL3 knockdown in T24 cells was restored following PKM2 overexpression, indicating recovery of glycolytic activity (**Figure 7G**). qPCR analysis confirmed efficient UCHL3 knockdown and PKM2 overexpression in both T24 and 5637 cells (**Figures 7H, I**). Collectively, these findings indicate that PKM2 overexpression largely counteracts the suppressive effects of UCHL3 knockdown on BCa cell proliferation, migration, and glycolytic metabolism, supporting PKM2 as an important downstream mediator of UCHL3 function in BCa cells.

**Figure 7:**
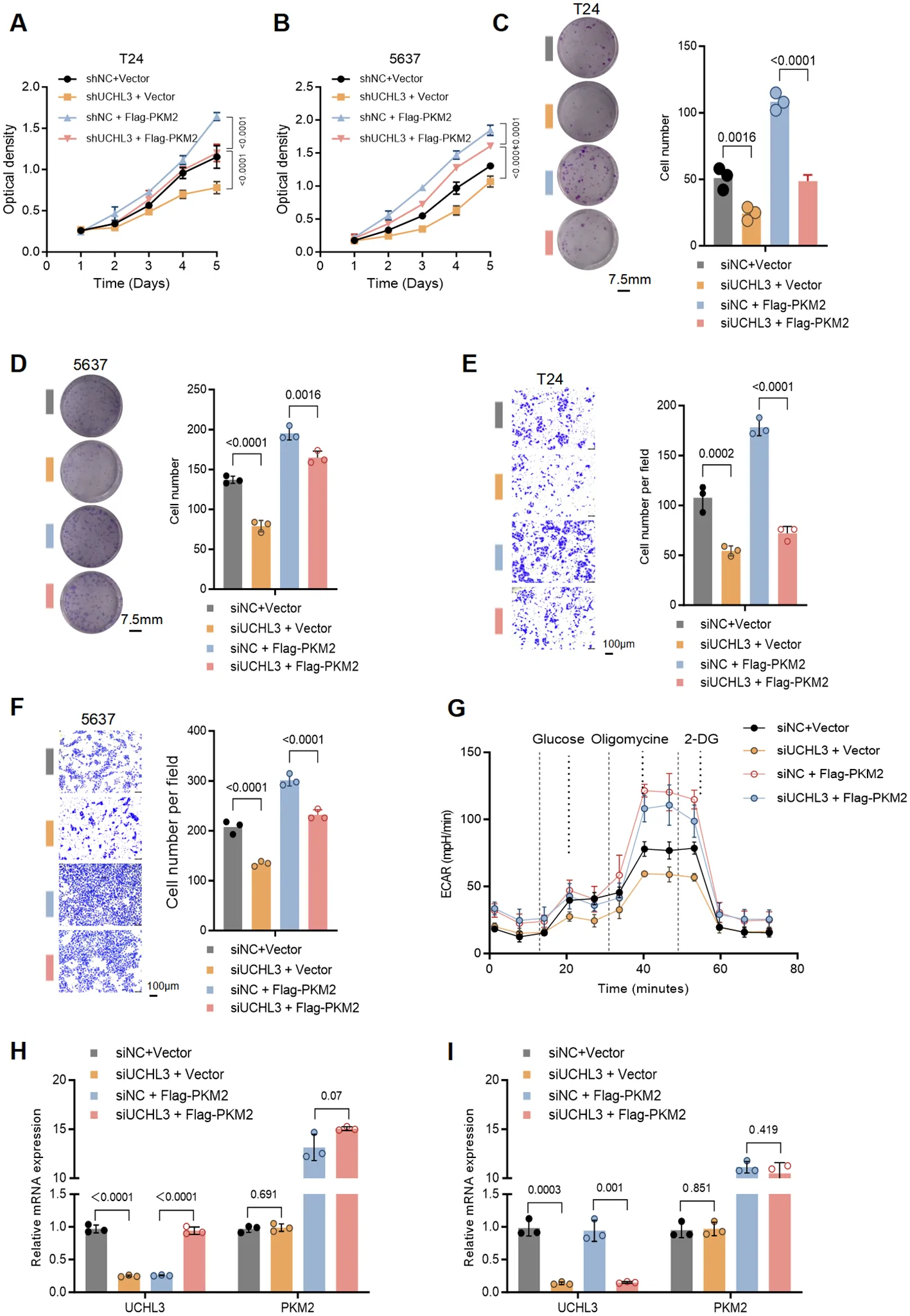
PKM2 overexpression rescues the inhibitory effects of UCHL3 knockdown on BCa cells. **(A, B)** Proliferation of T24 and 5637 cells with UCHL3 knockdown and PKM2 overexpression was assessed using the MTT assay (n = 5 per group). **(B, C)** Colony formation assays and statistical analysis were used to evaluate the proliferation of UCHL3-knockdown cells overexpressing PKM2 (n = 3 per group). **(E, F)** Transwell assay results indicating that PKM2 overexpression reverses UCHL3 knockdown-driven migration in T24 and 5637 cells (n = 3 per group). **(G)** Changes in cellular ECAR measured by the Seahorse XF Glycolysis Stress Test in UCHL3-knockdown T24 cells overexpressing PKM2 (n = 6 per group). **(H, I)** qPCR analysis of UCHL3 and PKM2 mRNA levels in T24 **(H)** and 5637 **(I)** cells following UCHL3 knockdown or PKM2 overexpression (n = 3 per group).

Taken together, these findings demonstrate that UCHL3 plays a critical role in promoting glycolysis in bladder cancer cells. Mechanistically, our data support a model in which UCHL3 enhances BCa glycolysis, tumor growth, and malignant progression by stabilizing PKM2 through deubiquitination and protecting it from proteasomal degradation. By preserving PKM2 protein abundance, UCHL3 likely facilitates the maintenance of a glycolytic metabolic program that supports the energetic and anabolic demands of bladder cancer progression (**Figure 8**).

**Figure 8:**
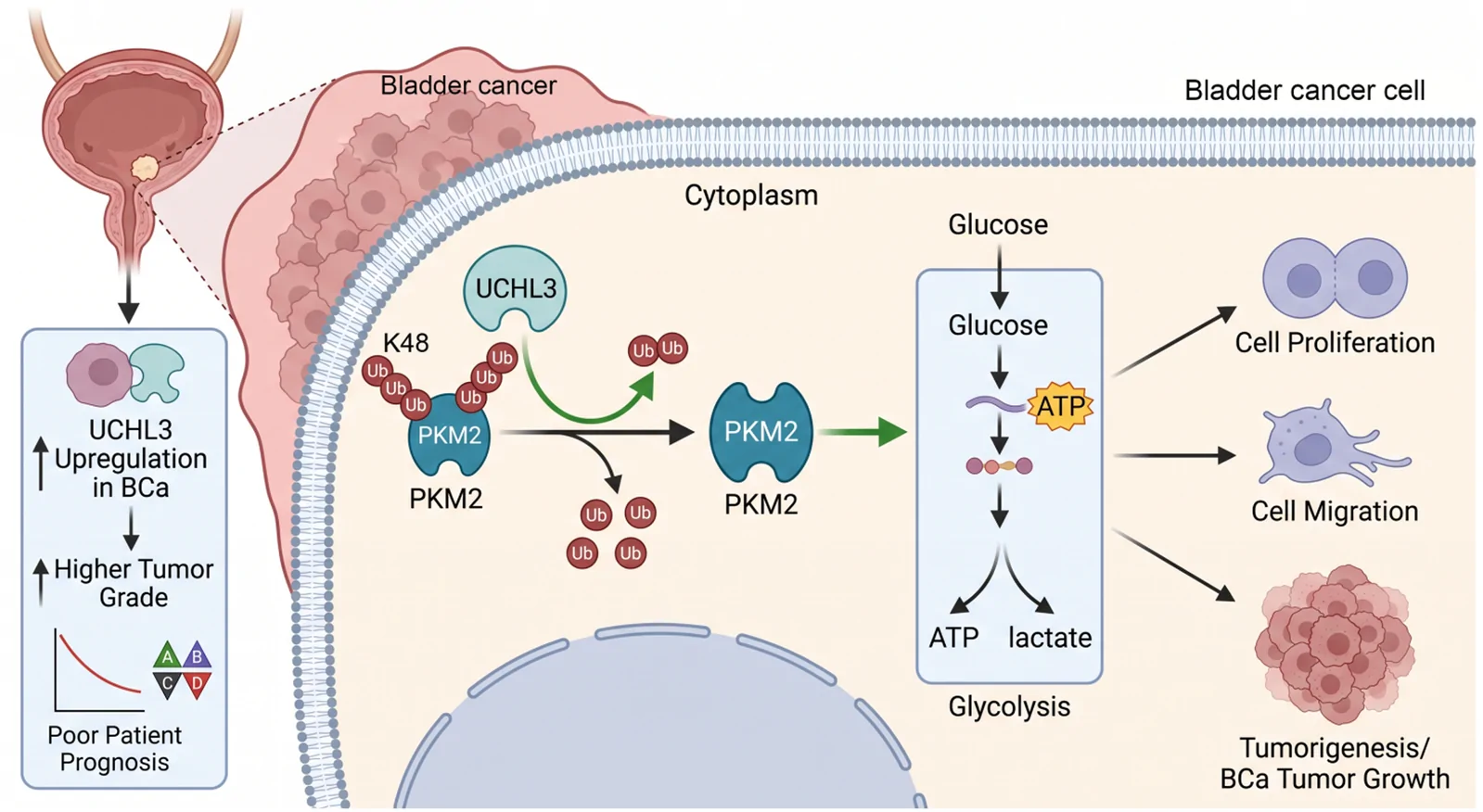
Mechanism diagram of the study. The schematic illustrates positive regulatory mechanism of UCHL3 on PKM2. UCHL3 can interact with PKM2 and undergo K48-linked deubiquitination of PKM2. This leads to the stability of PKM2 protein through the deubiquitination and protecting it from proteasomal degradation. By preserving PKM2 protein abundance, UCHL3 likely facilitates the maintenance of a glycolytic metabolic program that supports the energetic and anabolic demands of bladder cancer progression.

## Discussion

Bladder cancer (BCa) represents one of the most aggressive solid malignancies, yet the molecular underpinnings governing its initiation, progression, and metabolic reprogramming remain incompletely elucidated. As a core component of cancer metabolism, glycolysis is governed by multiple glycolytic enzymes, including hexokinase 2 (HK2), pyruvate kinase M2 (PKM2) (16), lactate dehydrogenase A (LDHA) (17), and phosphofructokinase 1 (PFK1) (18). Among these, PKM2 stands out due to its multifaceted oncogenic roles: elevated expression not only amplifies glycolytic flux to fuel the Warburg effect—enabling rapid ATP production, biomass synthesis, and lactate accumulation—but also interfaces with diverse non-metabolic signaling cascades, including STAT3, HIF-1α, and β-catenin pathways (5, 19–21). This dual functionality promotes hallmarks of cancer such as uncontrolled proliferation, evasion of apoptosis, epithelial-mesenchymal transition (EMT)-driven migration and invasion, and resistance to chemotherapeutic agents like cisplatin across a spectrum of tumors, including BCa (22). Consequently, PKM2 has long been heralded as a promising therapeutic vulnerability, with inhibitors such as shikonin or TLN-232 demonstrating preclinical efficacy, albeit often limited by off-target effects or compensatory metabolic adaptations (23).

Post-translational modifications, particularly ubiquitination, serve as a critical rheostat for protein homeostasis, dictating turnover, localization, and activity of key oncoproteins (24, 25). E3 ubiquitin ligases mark substrates for proteasomal degradation, while deubiquitinases (DUBs) counteract this process, conferring stability and functionality (26, 27). Dysregulation of the E3-DUB axis is a common phenomenon during tumorigenesis; however, research regarding how DUBs promote the stability of glycolytic enzymes in bladder cancer (BCa) remains limited. To bridge this gap, our DUB siRNA library screen, using PKM2 protein abundance as a primary endpoint, pinpointed several candidates, including UCHL3 and USP28. Notably, siRNA-mediated USP28 depletion elicited only marginal effects on PKM2 levels in our BCa models, potentially attributable to cell-line-specific regulatory contexts or compensatory mechanisms observed in other systems. This highlights the context-dependent nature of DUB-substrate interactions, underscoring the necessity for tissue-specific validation.

To define how individual DUB family members influence PKM2 deubiquitination and malignant phenotypes, a DUB siRNA library was screened with PKM2 protein abundance as the primary readout, yielding several candidate regulators. In addition to UCHL3, other enzymes such as USP28 emerged as potential modulators; however, siUSP28 produced minimal changes in PKM2 levels in this screening context, potentially reflecting cell line–dependent regulatory differences (28). Because only a subset of the DUB library was examined, additional PKM2-regulating DUBs may remain unidentified and require comprehensive screening. UCHL3, a member of the ubiquitin C-terminal hydrolase (UCH) subfamily, emerges as a particularly compelling regulator. It is frequently overexpressed in diverse malignancies—including lung adenocarcinoma, breast cancer, cholangiocarcinoma, gastric carcinoma, and melanoma—where it correlates with accelerated proliferation, metastatic dissemination, and poor clinical prognosis (26, 29). Mechanistically, UCHL3 exerts pleiotropic effects: beyond direct substrate deubiquitination, it remodels the inflammatory tumor microenvironment (TME) by suppressing anti-tumor immune responses, thereby attenuating efficacy of PD-1/PD-L1 blockade immunotherapy (30). Paradoxically, in certain contexts, UCHL3 can dampen NF-κB signaling, thereby upregulating MHC class I expression and priming innate immunity (31). Furthermore, UCHL3 stabilizes aryl hydrocarbon receptor (AhR), promoting tumorigenesis; its depletion via shRNA, CRISPR, or pharmacological inhibitors (e.g., TCID) triggers AhR proteasomal degradation and curtails proliferation (32).

In the present study, we establish UCHL3 as a bona fide deubiquitinase for PKM2 specifically in BCa. Co-immunoprecipitation assays confirmed a direct physical interaction, while functional assays demonstrated that UCHL3 depletion profoundly reduces PKM2 protein levels—a phenotype rescued exclusively by wild-type (WT) UCHL3 but not the catalytically inactive C95A mutant, affirming enzymatic dependence (33). Cycloheximide chase experiments revealed accelerated PKM2 turnover upon UCHL3 loss, with half-life shortening from approximately 8–12 hours to under 4 hours in representative cell lines. The gain-of-function CHX assay was performed in 5637 cells, and confirmation in low-UCHL3 RT-4 cells is warranted. Ubiquitination assays further showed that UCHL3-WT overexpression markedly attenuates K48-linked polyubiquitin chains on PKM2, whereas C95A fails to do so. Crucially, proteasome inhibition with MG132 abrogated the destabilizing effect of UCHL3 knockdown, confirming that regulation occurs via the ubiquitin-proteasome system rather than alternative degradative pathways (e.g., autophagy). These molecular insights translate directly to metabolic reprogramming: UCHL3 overexpression enhanced extracellular acidification rate (ECAR), glucose uptake, and lactate production—hallmarks of aerobic glycolysis—while PKM2 pharmacological inhibition (e.g., with compound 3K) or siRNA completely abolished these gains, positioning PKM2 as the obligate downstream effector.

These rescue experiments support PKM2 as an important functional downstream mediator of UCHL3 in BCa cells. Whether PKM2 similarly mediates the effect of UCHL3 on tumor growth *in vivo* remains to be determined using dedicated *in vivo* rescue models. This UCHL3-PKM2 axis integrates seamlessly with broader cancer metabolism paradigms. In BCa, where glycolysis sustains hypoxic adaptation and chemoresistance, our findings echo yet extend observations from other malignancies. For instance, analogous DUB-mediated PKM2 stabilization (e.g., via OTUB2 in colorectal cancer) similarly fuels glycolytic flux and EMT (34); notably, a parallel mechanism was recently delineated in hepatocellular carcinoma, wherein UCHL3 deubiquitinates PKM2 at lysine 206 to modulate cuproptosis sensitivity alongside glycolysis and HIF-1 signaling (35). Such conservation implies evolutionary pressure for DUBs to safeguard PKM2 in metabolically demanding tumor niches, though substrate specificity and downstream outcomes appear tissue-contextual—glycolysis-dominant in BCa versus cuproptosis-augmented in liver cancer (35). Edge cases warrant consideration: in nutrient-replete versus glucose-starved microenvironments, UCHL3-PKM2 stabilization may differentially influence tetramer (high-activity) versus dimer (nuclear-transcriptional) PKM2 forms, potentially linking metabolism to epigenetic regulation via histone phosphorylation or HIF-1α co-activation. Moreover, potential crosstalk with other UCHL3 substrates (e.g., β-catenin or AhR) could amplify feed-forward loops: stabilized PKM2 may enhance β-catenin nuclear translocation, further driving glycolytic gene transcription and creating a vicious cycle in BCa progression (32, 36).

In summary, our work delineates a novel UCHL3-PKM2 deubiquitination axis that stabilizes PKM2, fuels glycolysis, and propels BCa aggressiveness. By bridging ubiquitination dynamics with metabolic reprogramming, this mechanism not only refines our understanding of BCa pathogenesis but also nominates UCHL3 as a multifaceted therapeutic target. Future investigations should encompass *in vivo* genetic models, multi-omics integration (proteomics, metabolomics, single-cell RNA-seq), and early-phase trials to fully harness this pathway’s translational potential, ultimately advancing precision oncology for this recalcitrant disease.

## Data Availability

The raw data supporting the conclusions of this article will be made available by the authors, without undue reservation.
