# Supplementary material for: The deubiquitinating enzyme UCHL3 promotes bladder cancer progression and glycolysis through enhancing PKM2 protein stability
Source: Front Oncol. 2026 Sep 18;16:1883763. doi: 10.3389/fonc.2026.1883763 (PMC13630518; doi:10.3389/fonc.2026.1883763)

Figure 1A

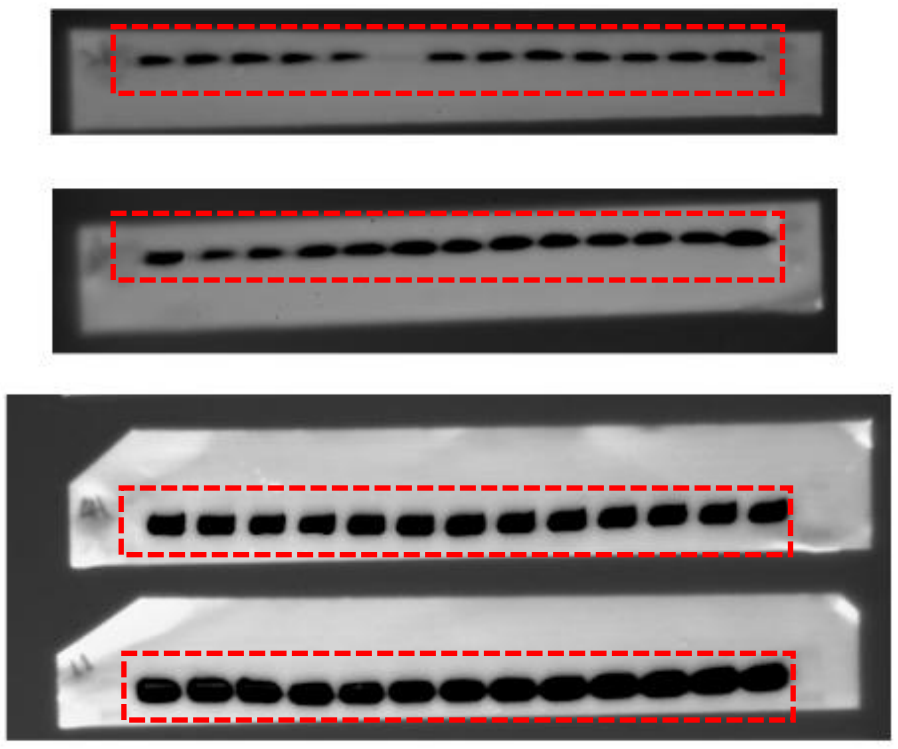

Figure 1C

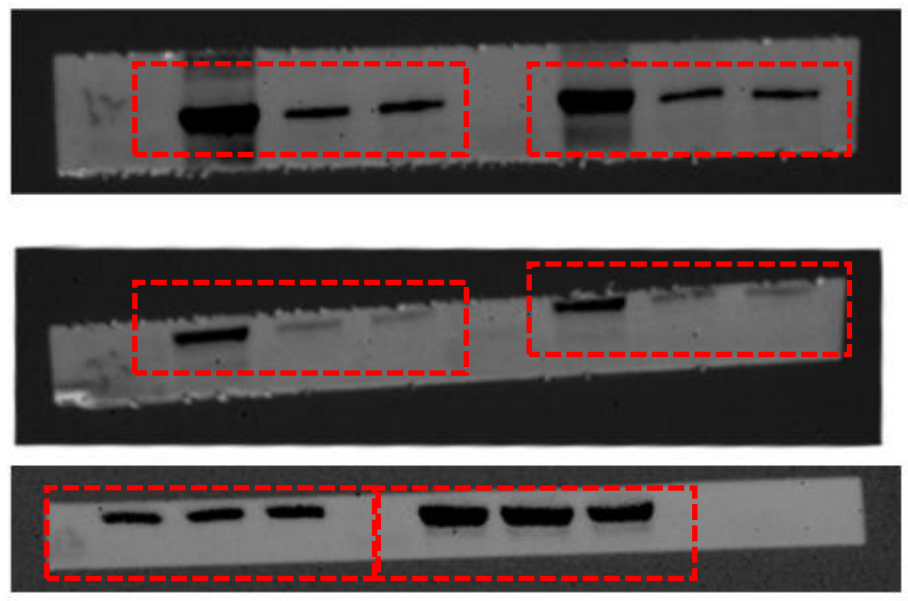

Figure 1F

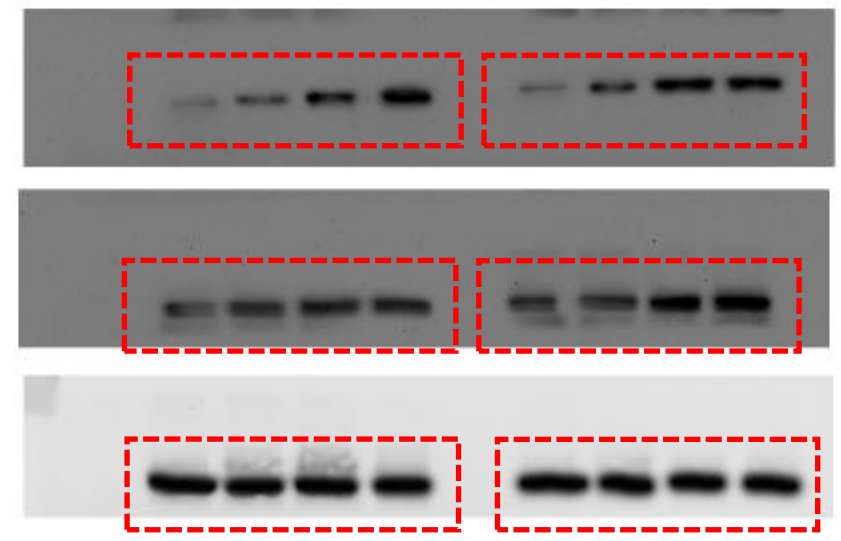

Figure 2A

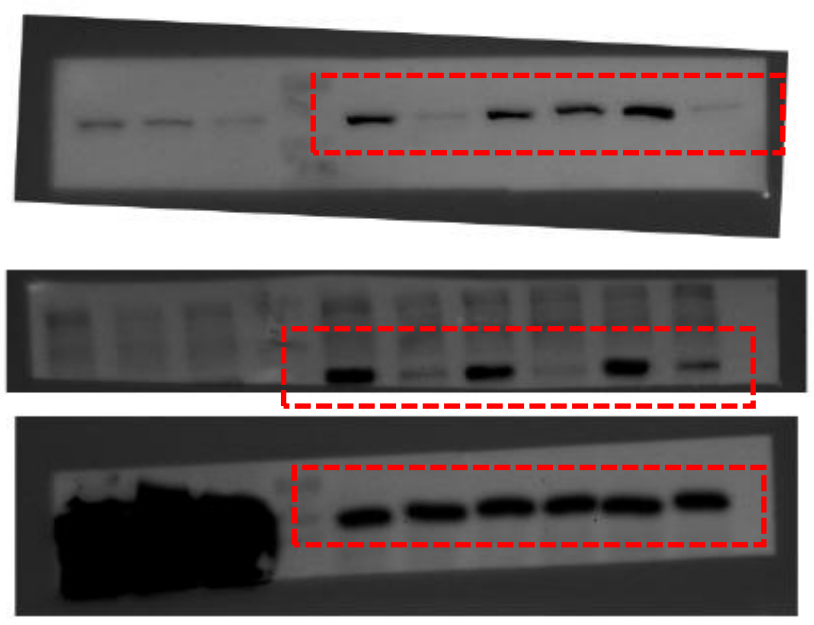

Figure 2B

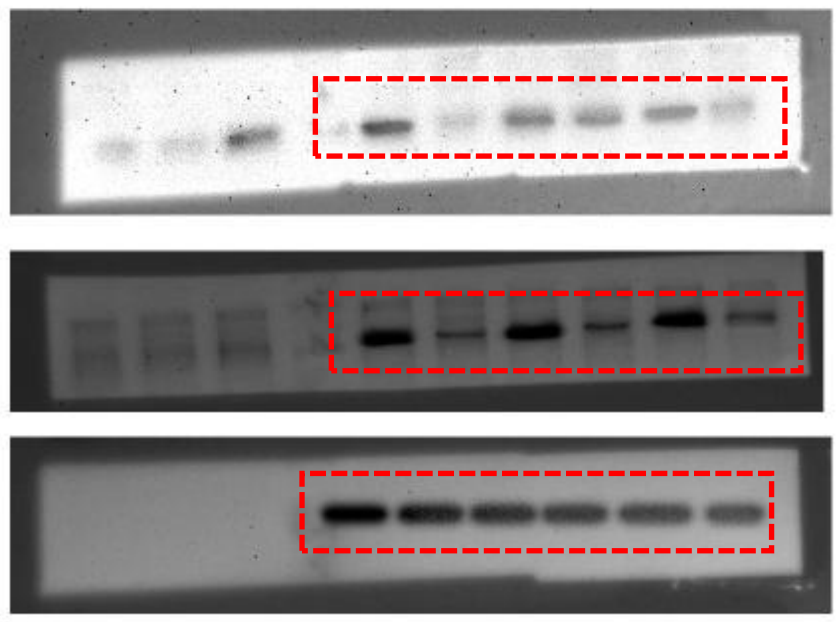

Figure 2C-D

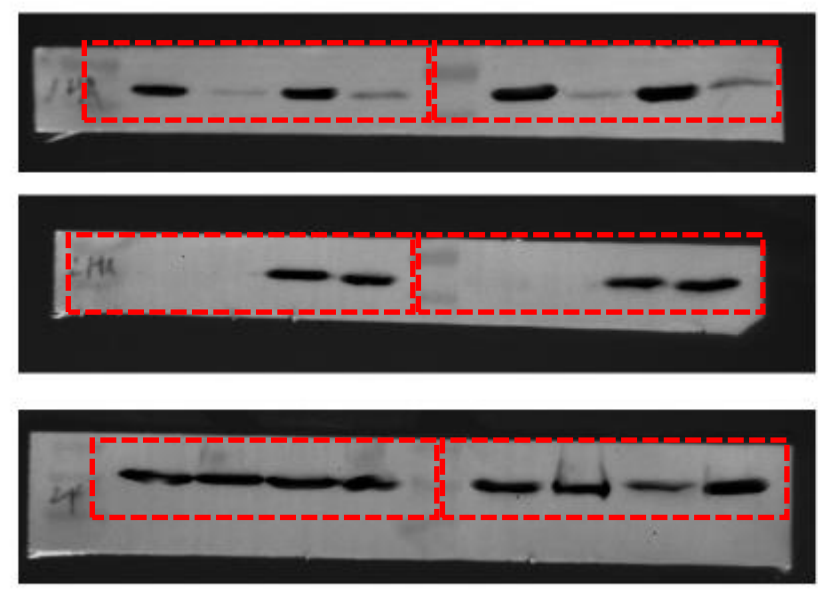

Figure 2E

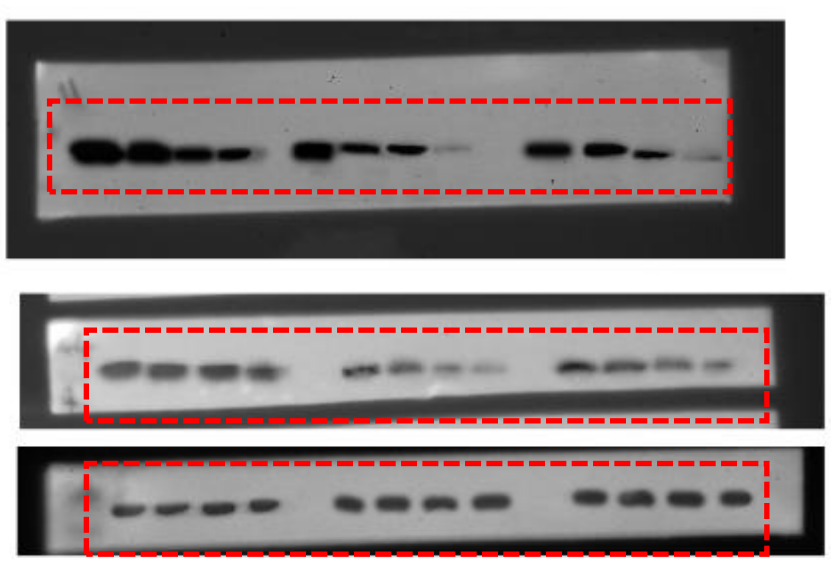

Figure 2F

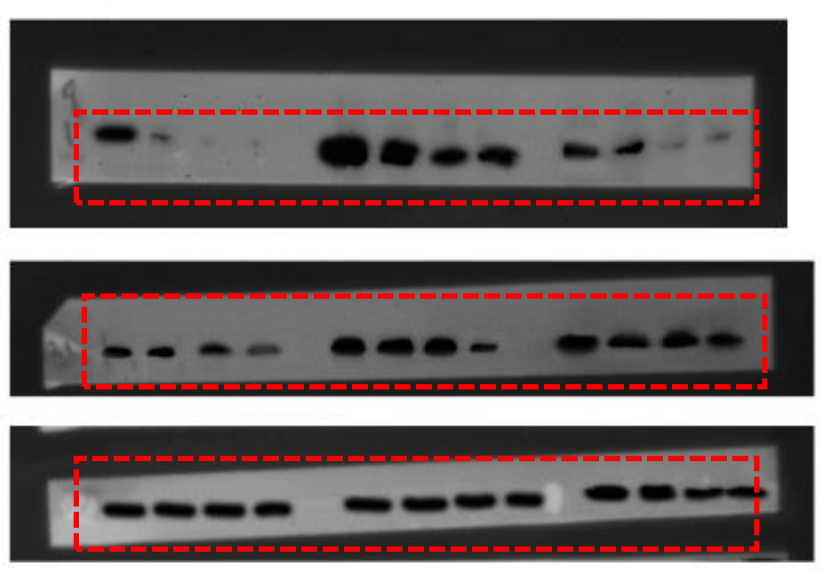

Figure 3B

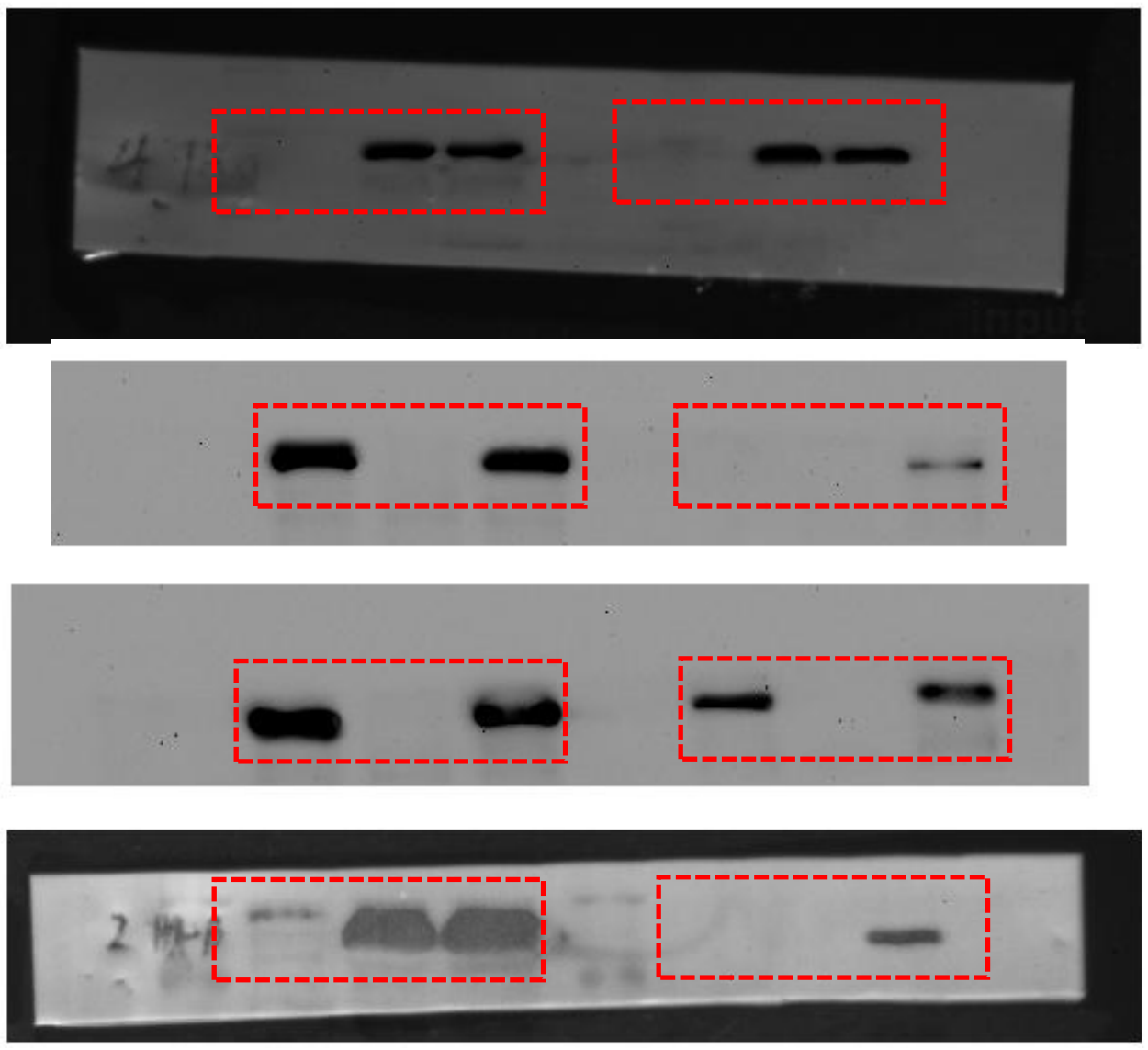

Figure 3C

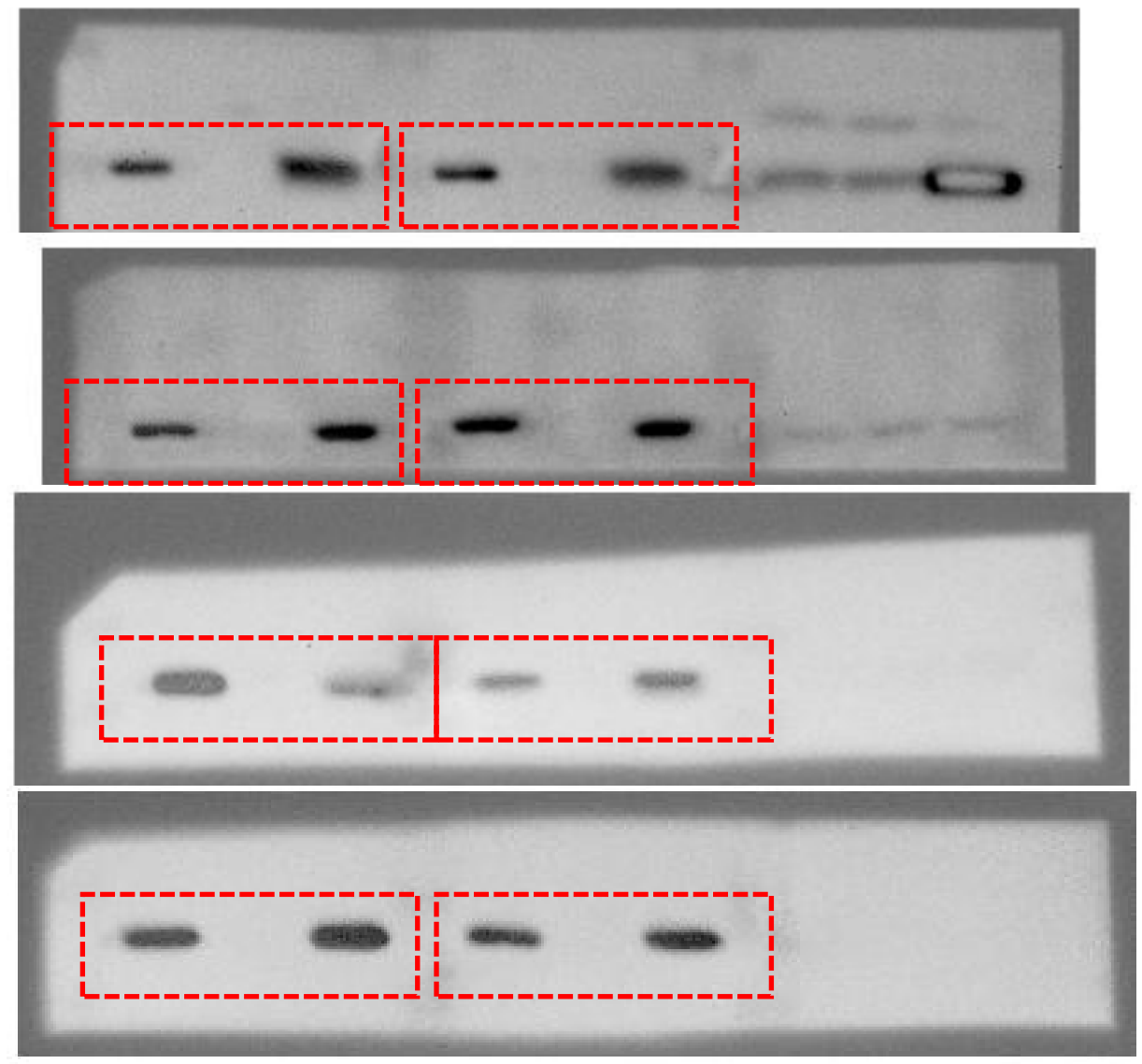

Figure 3D-E

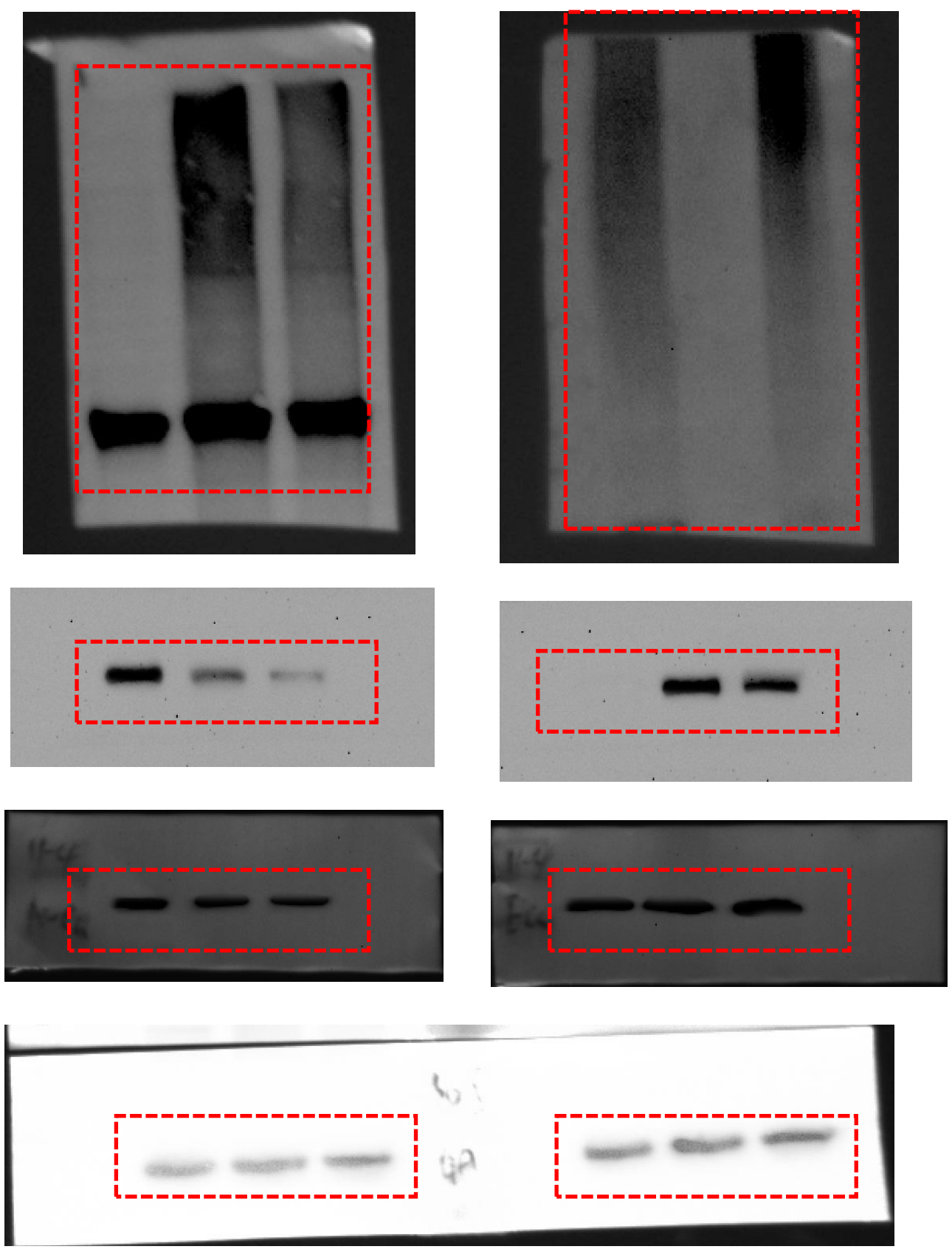

Figure 3F

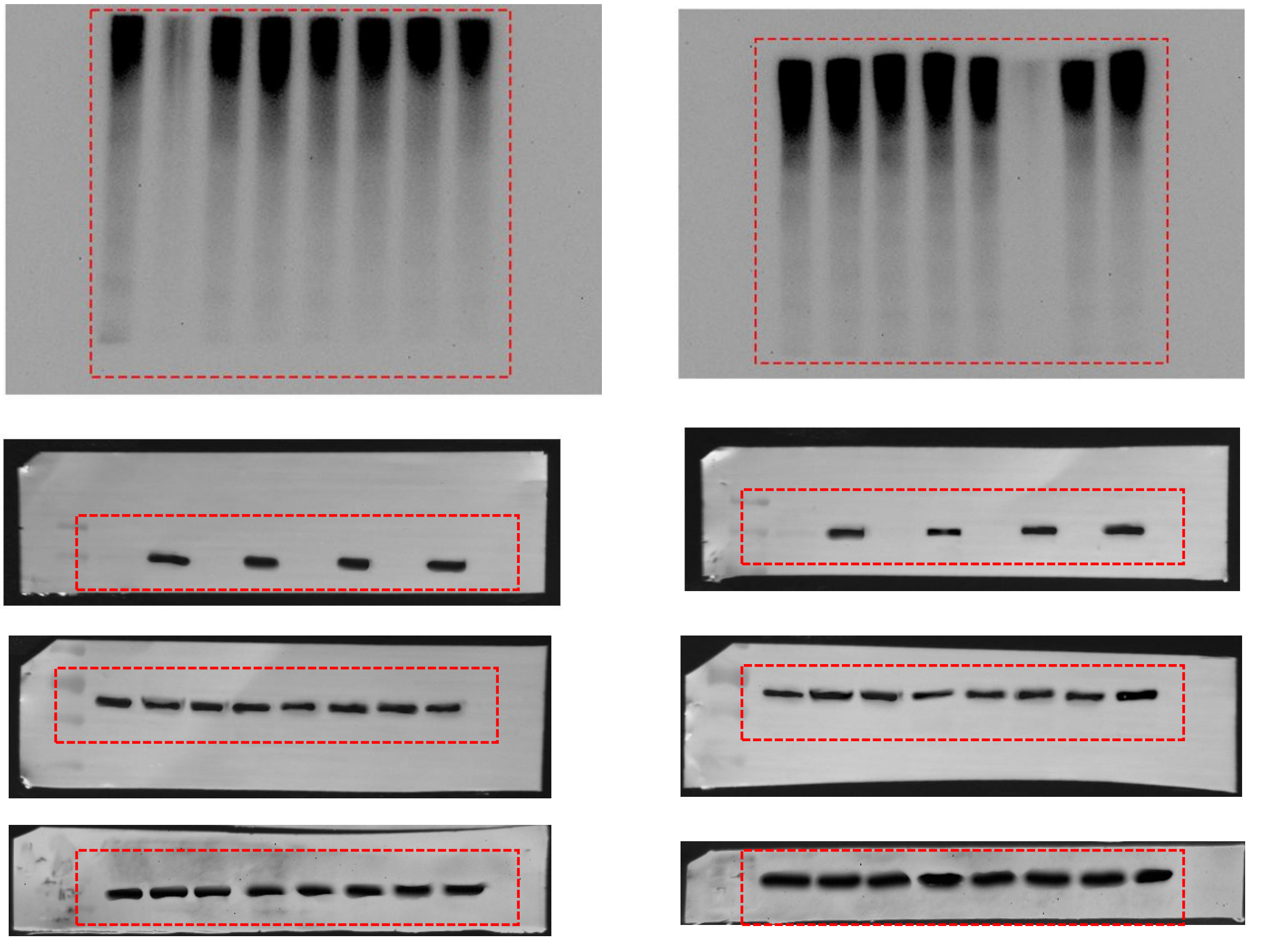

Supplement: Supplementary file 1 [file DataSheet1.pdf]
