# Supplementary material for: The deubiquitinating enzyme UCHL3 promotes bladder cancer progression and glycolysis through enhancing PKM2 protein stability
Source: Front Oncol. 2026 Sep 18;16:1883763. doi: 10.3389/fonc.2026.1883763 (PMC13630518; doi:10.3389/fonc.2026.1883763)
Supplement: Supplementary file 2 [file SupplementaryFile1.docx]

**Supplement materials**

**Supplementary Table S1: The sequences of UCHL3 siRNAs and PKM2 siRNAs used in this study.**

| **Gene** | **Sequences for siRNA** |
| --- | --- |
| Control siRNA | Sense: 5′-UUCUCCGAACGUGUCACGUTT-3′ |
| UCHL3 siRNA-1 | Sense: 5′-GGCGGAAGCCAUUUCCAAUTT-3′ |
| UCHL3 siRNA-2 | Sense: 5′-CCCUGAUGAACUAAGAUUUTT-3′ |
| PKM2 siRNA | Sense: 5′-GGATGTTGATATGGTGTTT-3′ |

**Supplementary Table S2: The specific information for the primary antibodies is as follows:**

| **Primary antibodies** | **Details** |
| --- | --- |
| UCHL3 | 1:1000, 12384-1-AP, proteintech, China |
| UCHL3 | 1:200, sc-100340, Santa Cruz Biotech., CA, USA |
| PKM2 | 1:10000, 15822-1-AP, proteintech, China |
| GAPDH | 1:15000, 60004-1-Ig, proteintech, China |
| β-actin | 1:1000, sc-58673, Santa Cruz Biotech., CA, USA |
| ubiquitin | 1:1000, 10201-2-AP, proteintech, China |
| Flag tag | 1:1000, 66008-4-Ig, proteintech, China |
| Myc tag | 1:1000, AE070, ABclonal, China |
| lgG | 1:1000, 30000-0-AP, proteintech, China |
| HA tag | 1:5000, M20021, abmart, Shanghai, China |
| HA tag | 1:1000, #561,MBL, Nagoya, Japan |

| **Gene** | **Primer sequences (5'-3')** | **Annealing temperature(℃)** |
| --- | --- | --- |
| UCHL3 | Forward: GGAAGAGTCCAAGCGTGAGG | 60 |
|  | Reverse: GAAACTGGTTGGTGACCCTCT |  |
| PKM2 | Forward: ATGTCGAAGCCCCATAGTGAA | 59 |
|  | Reverse: TGGGTGGTGAATCAATGTCCA |  |
| GAPDH | Forward: 5’-CACCCACTCCTCCACCTTTG-3’ | 60 |
|  | Reverse: 5’-CCACCACCCTGTTGCTGTAG-3’ |  |

**Supplementary Table S3. Primer sequences used in qRT-PCR assays.**

**Supplementary Table S4. Complete list and quantitative results of the exploratory siRNA screen of 24 DUBs for regulators of PKM2 protein abundance.** PKM2 band intensities were normalized to β-actin. Within each replicate experiment, the normalized value for each siDUB condition was subsequently divided by the corresponding siControl value, with siControl set to 1.0. Data are presented as individual replicate values and mean ± SD. The initial screen was exploratory and hypothesis-generating; no formal numerical cutoff was prespecified. UCHL3 was prioritized because its depletion resulted in the strongest and most reproducible reduction in PKM2 abundance and was subsequently validated using two independent siRNAs in T24 and 5637 cells.

| **No.** | **Gene symbol** | **β-actin-normalized Rep 1** | **Rep 2** | **Rep 3** | **Relative PKM2 Rep 1** | **Rep 2** | **Rep 3** | **Mean relative PKM2** | **SD** | **Change vs siControl (%)** | **Screening interpretation** |
| --- | --- | --- | --- | --- | --- | --- | --- | --- | --- | --- | --- |
| 1 | UFSP2 | 1.071293 | 1.167197 | 1.120132 | 1.142834 | 1.113309 | 1.104451 | 1.120198 | 0.020098 | 12.02 | Not prioritized |
| 2 | SENP8 | 1.078604 | 1.042115 | 0.943774 | 1.150634 | 0.994002 | 0.930562 | 1.025066 | 0.113277 | 2.51 | Not prioritized |
| 3 | SENP1 | 0.966539 | 0.85685 | 0.878404 | 1.031084 | 0.817291 | 0.866107 | 0.904827 | 0.112033 | -9.52 | Not prioritized |
| 4 | USP16 | 0.845782 | 0.94298 | 1.047886 | 0.902264 | 0.899444 | 1.033216 | 0.944975 | 0.076432 | -5.50 | Not prioritized |
| 5 | UCHL3 | 0.253792 | 0.280037 | 0.25501 | 0.270741 | 0.267108 | 0.251440 | 0.263096 | 0.010256 | -73.69 | Prioritized candidate; strong and reproducible PKM2 reduction |
| 6 | JOSD1 | 1.095759 | 0.967585 | 1.012127 | 1.168934 | 0.922913 | 0.997958 | 1.029935 | 0.126089 | 2.99 | Not prioritized |
| 7 | UFSP1 | 1.097198 | 1.028291 | 0.958318 | 1.170469 | 0.980817 | 0.944902 | 1.032063 | 0.121201 | 3.21 | Not prioritized |
| 8 | JOSD2 | 1.117386 | 1.2496 | 1.028498 | 1.192006 | 1.191908 | 1.014100 | 1.132671 | 0.102686 | 13.27 | Not prioritized |
| 9 | UBTD1 | 0.931266 | 1.022626 | 0.804892 | 0.993456 | 0.975413 | 0.793624 | 0.920831 | 0.110533 | -7.92 | Not prioritized |
| 10 | UBL4A | 0.772654 | 0.725065 | 0.771036 | 0.824252 | 0.691590 | 0.760242 | 0.758695 | 0.066345 | -24.13 | Not prioritized |
| 11 | USP2 | 0.9653 | 0.959565 | 0.881603 | 1.029763 | 0.915264 | 0.869261 | 0.938096 | 0.082651 | -6.19 | Not prioritized |
| 12 | UBL3 | 0.947253 | 0.940843 | 0.975616 | 1.010511 | 0.897406 | 0.961958 | 0.956625 | 0.056741 | -4.34 | Not prioritized |
| 13 | USP26 | 0.7193 | 0.782645 | 0.704068 | 0.767335 | 0.746512 | 0.694212 | 0.736019 | 0.037674 | -26.40 | Not prioritized |
| 14 | USP21 | 0.825959 | 0.897395 | 0.894777 | 0.881117 | 0.855964 | 0.882251 | 0.873111 | 0.014860 | -12.69 | Not prioritized |
| 15 | USP5 | 0.910152 | 0.947539 | 0.823962 | 0.970932 | 0.903793 | 0.812427 | 0.895717 | 0.079561 | -10.43 | Not prioritized |
| 16 | OTUB1 | 0.86586 | 0.877241 | 0.730058 | 0.923683 | 0.836740 | 0.719838 | 0.826754 | 0.102289 | -17.32 | Not prioritized |
| 17 | UCHL5 | 0.949948 | 0.971259 | 0.900358 | 1.013386 | 0.926418 | 0.887754 | 0.942519 | 0.064345 | -5.75 | Not prioritized |
| 18 | USP9Y | 0.877767 | 0.876299 | 0.85742 | 0.936385 | 0.835842 | 0.845417 | 0.872548 | 0.055491 | -12.75 | Not prioritized |
| 19 | OTUD4 | 0.915009 | 0.885393 | 0.95322 | 0.976114 | 0.844516 | 0.939876 | 0.920168 | 0.067976 | -7.98 | Not prioritized |
| 20 | USP9X | 0.88916 | 0.865605 | 0.82389 | 0.948539 | 0.825641 | 0.812356 | 0.862179 | 0.075084 | -13.78 | Not prioritized |
| 21 | OTUD1 | 0.828125 | 0.81282 | 0.773349 | 0.883428 | 0.775293 | 0.762523 | 0.807081 | 0.066425 | -19.29 | Not prioritized |
| 22 | OTUD5 | 0.99732 | 1.001956 | 0.965347 | 1.063921 | 0.955697 | 0.951833 | 0.990484 | 0.063628 | -0.95 | Not prioritized |
| 23 | USP28 | 0.887164 | 0.911437 | 0.877762 | 0.946409 | 0.869357 | 0.865474 | 0.893747 | 0.045648 | -10.63 | Secondary candidate; marginal PKM2 reduction |
| 24 | USP54 | 1.103246 | 1.098791 | 1.041916 | 1.176921 | 1.048062 | 1.027330 | 1.084104 | 0.081047 | 8.41 | Not prioritized |

Notes:

1. The supplied densitometry values were normalized to β-actin.

2. For each biological replicate, the β-actin-normalized value of each siDUB condition was divided by the matched siControl value; therefore, siControl = 1 within each replicate.

3. Change vs siControl (%) = (mean relative PKM2 − 1) × 100.

4. The initial screen was exploratory and hypothesis-generating; no post hoc numerical cutoff should be introduced unless it was genuinely prespecified.

5. UCHL3 was subsequently validated with two independent siRNAs in T24 and 5637 cells, as described in the main manuscript.

**Figure S1.** Expression of UCHL3 in BCa of TCGA samples. (A) Expression of UCHL3 in different tumor types from the TIMER database. (B) UCHL3 expression in the normal and primary tumor of the bladder. (C) Expression of UCHL3 in BCa based on tumor stage. (D) UCHL3 expression is based on different histological subtypes: papillary tumor and non-papillary tumor. (E) Expression of UCHL3 in BCa based on node metastasis status. ***p < 0.001, **p < 0.01, *p < 0.05.

**Figure S2.** PKM2 is overexpressed in BCa tissues and is associated with unfavorable prognosis of patients with BCa. (A) PKM2 expression in the normal and primary tumor of the bladder. (B) Expression of PKM2 in BCa based on tumor stage. (C) PKM2 expression is based on different histological subtypes: papillary tumor and non-papillary tumor. (D) Expression of PKM2 in BCa based on node metastasis status. (E) Kaplan-Meier survival analysis regarding PKM2 in BCa patients based on the Ualcan database (https://ualcan.path.uab.edu). ***p < 0.001, **p < 0.01, *p < 0.05.
